# Improving TB detection among children in routine clinical care through intensified case finding in facility-based child health entry points and decentralized management: A before-and-after study in Nine Sub-Saharan African Countries

**DOI:** 10.1371/journal.pgph.0002865

**Published:** 2024-02-05

**Authors:** Jean-François Lemaire, Jennifer Cohn, Shirin Kakayeva, Boris Tchounga, Patricia Fassinou Ekouévi, Vicky Kambaji Ilunga, Donald Ochieng Yara, Samson Lanje, Yusuf Bhamu, Leo Haule, Mary Namubiru, Tichaona Nyamundaya, Maude Berset, Mikhael de Souza, Rhoderick Machekano, Martina Casenghi, the CaP-TB Study team

**Affiliations:** 1 Elizabeth Glaser Pediatric AIDS Foundation, Geneva, Switzerland; 2 Division of Infectious Diseases, University of Pennsylvania School of Medicine, Philadelphia, Pennsylvania, United States of America; 3 Elizabeth Glaser Pediatric AIDS Foundation, Washington, District of Columbia, United States of America; 4 Elizabeth Glaser Pediatric AIDS Foundation, Yaounde, Cameroon; 5 Elizabeth Glaser Pediatric AIDS Foundation, Abidjan, Côte d’Ivoire; 6 Elizabeth Glaser Pediatric AIDS Foundation, Kinshasa, République Démocratique du Congo; 7 Elizabeth Glaser Pediatric AIDS Foundation, Nairobi, Kenya; 8 Elizabeth Glaser Pediatric AIDS Foundation, Maseru, Lesotho; 9 Elizabeth Glaser Pediatric AIDS Foundation, Lilongwe, Malawi; 10 Elizabeth Glaser Pediatric AIDS Foundation, Dar es Salaam, Tanzania; 11 Elizabeth Glaser Pediatric AIDS Foundation, Kampala, Uganda; 12 Elizabeth Glaser Pediatric AIDS Foundation, Harare, Zimbabwe; Boston University, UNITED STATES

## Abstract

In 2022, an estimated 1.25 million children <15 years of age developed tuberculosis (TB) worldwide, but >50% remained undiagnosed or unreported. WHO recently recommended integrated and decentralized models of care as an approach to improve access to TB services for children, but evidence remains limited. The Catalyzing Paediatric TB Innovation project (CaP-TB) implemented a multi-pronged intervention to improve TB case finding in children in nine sub-Saharan African countries. The intervention introduced systematic TB screening in different facility-based child-health entry-points, decentralisation of TB diagnosis and management, improved sample collection with access to Xpert^®^ MTB/RIF or MTB/RIF Ultra testing, and implementation of contact investigation. Pre-intervention records were compared with those during intervention to assess effect on paediatric TB cascade of care. The intervention screened 1 991 401 children <15 years of age for TB across 144 health care facilities. The monthly paediatric TB case detection rate increased significantly during intervention versus pre-intervention (+46.0%, 95% CI 36.2–55.8%; p<0.0001), with variability across countries. The increase was greater in the <5 years old compared to the 5–14 years old (+53.4%, 95% CI 35.2–71.9%; p<0.0001 versus +39.9%, 95% CI 27.6–52.2%; p<0.0001). Relative contribution of lower-tier facilities to total case detection rate increased from 37% (71.8/191.8) pre-intervention to 50% (139.9/280.2) during intervention. The majority (89.5%) of children with TB were identified through facility-based intensified case-finding and primarily accessed care through outpatient and inpatient departments. In this multi-country study implemented under real-life conditions, the implementation of integrated and decentralized interventions increased paediatric TB case detection. The increase was driven by lower-tier facilities that serve as the primary point of healthcare contact for most patients. The effect was greater in children < 5 years compared to 5–14 years old, representing an important achievement as the TB detection gap is higher in this subpopulation. (Study number NCT03948698)

## Introduction

Tuberculosis (TB) is a key cause of mortality in children, with most deaths occurring in those who were never diagnosed or received TB treatment [1]. Of the 1.25 million children and young adolescents <15 years of age estimated to fall ill with TB globally in 2022, only 49% were notified to national TB programmes, with only 42% reported among children 0−4 years [2]. Children under five years of age are more likely to be undiagnosed and have higher risk of developing active TB and severe disease, compared with older children [3]. TB is among the top ten causes of mortality in children 1–4 years old [4].

Common challenges in addressing the TB case finding gap, especially in children and young adolescents, have been well described [5, 6]. These include: poor prioritisation of paediatric and adolescent populations by TB programmes; reduced bacteriological confirmation compared to adults, especially in children 0–4 years, due to paucibacillary nature of TB disease; challenges in obtaining samples, suboptimal sensitivity of commercially available diagnostic assays; reliance on clinical diagnosis for which there is, limited expertise and confidence at lower-tier healthcare facilities such as primary health care facilities and district level hospital; weak integration of TB care into child health programmes [7–9]. Introducing systematic TB screening across different health facility services and strengthening TB services at the primary healthcare level can improve paediatric TB case finding in resource-limited settings [10, 11]. Integration of systematic TB screening in maternal and child health (MCH) services and strengthening of child contact investigation in urban health centres in Ethiopia [12], integration of TB screening in outpatients clinics, paediatric emergency rooms, and HIV clinics in Nigeria [13], implementation of community-based household contact investigations (HCI) and intensified paediatric TB case finding in outpatient clinics in Kenya [14], and decentralisation of child TB services in Uganda [15, 16], have been associated with improved paediatric TB case detection.

Integrated and decentralised service delivery approaches could play a key role in identifying missing paediatric TB cases [17–19]. The role of such interventions is emphasised in the latest World Health Organization (WHO) guidelines on management of TB in children and adolescents [20], but programmatic implementation has been slow, with many countries experiencing operational challenges [21, 22]. Symptomatic children with undiagnosed TB may access care through various healthcare entry points including general outpatient departments (OPD), inpatient departments (IPD), MCH, nutrition (NUT) and HIV services [23–27], but systematic TB screening is seldom performed at these services [27, 28]. Additionally, frontline healthcare workers’ (HCW) confidence in recognising childhood TB remains limited, especially in lower-level facilities, where the majority of children first access care [29].

The Catalyzing Paediatric TB Innovation project (CaP-TB) implemented a multi-pronged intervention designed to improve paediatric TB case finding in children and young adolescents <15 years of age across nine sub-Saharan African countries. We hypothesised that an intervention focusing on i) increasing the capacity of HCW to diagnose and treat paediatric TB at lower-level health facilities, ii) decentralizing the availability of specialised services such as sample collection procedures in peripheral sites, iii) providing systematic TB screening across different entry points and services usually attended by children, and iv) strengthening the implementation of HCI, would increase paediatric TB case detection and result in improved treatment success. Here we present findings from the CaP-TB intervention on the paediatric TB case detection and treatment cascade, and compare pre-intervention outcomes with those during intervention.

## Methods

### Study design and participants

Implementation of the CaP-TB project was led and supported by the Elizabeth Glaser Pediatric AIDS Foundation (EGPAF), in collaboration with the Ministry of Health (MoH) in each country. Facilities were selected from nine sub-Saharan African countries (Cameroon, Côte d’Ivoire, Democratic Republic of the Congo [DRC], Kenya, Lesotho, Malawi, Tanzania, Uganda, and Zimbabwe). As the CaP-TB intervention is an implementation programme carried out in close collaboration with MoHs in each country, randomisation was neither feasible nor pragmatic to use for the study and convenience sampling of facilities was applied.

A before-and-after study was implemented to evaluate the effect of project interventions. Analyses comparing pre-intervention with findings during intervention are restricted to 144 sites enrolled during the initial phase of the project, for which pre-intervention data could be collected. These sites were purposively selected to represent a range of healthcare settings (i.e. urban and rural settings, different healthcare system levels). Most (140/144) were district or primary-level facilities. The remaining four facilities were provincial/regional hospitals (2/144) and national reference hospitals (2/144). Facilities were categorized into lower -level (dispensaries, clinics, health centers), intermediate-level (small hospitals, including district level hospitals) and higher-level facilities (provincial, regional, or national level hospitals) based on standardized descriptions of facility services and health care staff cadres. Analyses of the impact of COVID-19 included all intervention sites enrolled as of July 2019 (N = 161), irrespective of availability of pre-intervention data.

The study population included all children aged 0−14 years presenting at study sites and household child contacts of TB index cases diagnosed at these sites. Pre-intervention data were retrospectively collected by EGPAF staff, and were extracted from MoH presumptive TB, TB treatment, IPT and contact investigation registers for all entries recorded over a complete 12-month period ending at least six months before the date of data extraction. This period varied per country, but overall was included between March 2017 and August 2018. The entry point variable was not systematically recorded in registers during pre-intervention. During intervention, children were consecutively enrolled from December 2018 to June 2021 and data were prospectively collected, using project-specific forms for screening as well as for investigation and treatment (individual forms were used for every child attended). The project forms were filled in by HCW at facilities and completion monitored by EGPAF staff during the regular support and supervision visits. Data recorded included TB symptoms, entry point, test result, and dates of sample collection and TB treatment initiation. Events not documented through those project-specific forms were not quantified and were excluded from analyses. TB treatment outcomes data were extracted from facility registers. De-identified baseline and intervention data were entered into a project-specific data management system.

This study is registered with ClinicalTrials.gov, number NCT03948698. Ethical approval was granted by Advarra (Pro00028743), WHO Ethics Review Committee (ERC) and by appropriate authorities in each country. A waiver for informed consent and for parents/guardians’ consent was granted for patient-level data collection by Advarra, WHO ERC, and in-country institutional review boards.

### Procedures

The CaP-TB intervention included: i) introduction of systematic and child-adapted symptom-based TB screening in entry points attended by children, often performed by community health workers (CHW); ii) HCW training on prevention, diagnosis and management of TB in children and young adolescents, including chest X-ray interpretation; iii) implementation of sample collection procedures, including expectorated sputum, induced sputum, nasopharyngeal aspirate, gastric aspirate, and fine-needle aspiration, depending on health facility infrastructure and national guidelines, including support for sample transportation to molecular testing sites when needed (Xpert^®^ MTB/RIF or MTB/RIF Ultra [Cepheid, Sunnyvale, USA], was used depending on country; see supporting information file for more details); iv) HCI, including both facility- and community-based approaches.

Paediatric TB screening was performed using a child-adapted list of signs and symptoms including: cough, fever, wheeze, night sweats (for young adolescents >10 years), fatigue or lethargy, loss of appetite or failure to thrive, neck swelling, history of close contact with a TB index patient during the last 12 months. Initial screening was performed at triage or in waiting areas by nurses or CHWs. Symptomatic children were further assessed by clinicians who were responsible to confirm whether or not a child had presumptive TB. Presumptive TB among HIV-positive children was defined as presence of any sign or symptom, irrespective of duration, or close contact with a TB index case. HIV-negative children were identified as having presumptive TB if any persistent (duration varied per country and ranged from >7 days to >14 days) sign or symptom was detected. Investigation of presumptive TB cases was performed according to national guidelines, access to technology, and manufacturer’s instructions. Children were defined as investigated for TB if they underwent either laboratory-based investigations and/or clinical assessment with or without chest X-ray investigation, and where the clinician documented such investigation using a project form. Not all sample collection techniques were available at all sites and depended on facility context and infrastructure. Form of TB (pulmonary versus extrapulmonary) and treatment success were defined according to national policies and WHO guidance. For further details, please see the supplementing information.

### Outcome measures

Outcome measures included number and proportion (overall and by entry point and country) of: i) children screened for TB; ii) screened children identified as presumptive TB; iii) children identified as presumptive TB investigated for TB; iv) investigated cases diagnosed with active TB; v) diagnosed children with bacteriologically confirmed TB; vi) diagnosed children initiated on TB treatment; vii) children initiated on treatment who achieved treatment success (as defined by WHO). The number needed to screen to detect one TB case (NNS) was also calculated.

Proportions for paediatric TB diagnosis and treatment outcomes were compared pre-intervention and during intervention. The effect of project interventions on paediatric TB case detection was additionally measured using the network monthly rate (NMR). The NMR is the mean number (+/- standard deviation) of 0–14 year old TB cases diagnosed monthly across all 144 sites combined. The enrolment of sites was progressive and therefore resulted in sites having different length of intervention exposure. The first month of intervention was defined as the first month during which all project sites were enrolled in a given country (timepoint varying in each country). Hence, the NMR allows to account for possible seasonal trends, as in this analysis, the first month of intervention corresponds to different calendar months in each of the country. The NMR calculated for the period during intervention includes children diagnosed with TB during the first 17 months of intervention in each country. Monthly time-series for the NMR were also produced to evaluate changes in NMR pre- and during intervention. In addition to overall NMR, data were disaggregated by age-bands (0–4 and 5–14 years), by country and healthcare facility level. As a sensitivity analysis, we also performed a site-level analysis comparing the medians of site-level monthly detection rates (Monthly Rate per Site, MRS) for children aged 0–14 years diagnosed with TB pre- and during intervention periods, and where the complete period of intervention for each specific site was used to determine their respective monthly rate (e.g. the total number of children 0–14 years diagnosed at a given site was divided by the number of months during which the intervention was implemented at the specific site)). The use of MRS allowed to assess shifts in the medians during intervention compared to pre-intervention, limiting the possible effect of intrinsic differences in the levels of contribution of sites due to their attendance, specialised services, or length of intervention.

The analysis of the impact of the COVID-19 pandemic on paediatric TB diagnosis during the intervention period was post hoc. For further details on the analysis and on the definitions of the Before COVID-19, COVID-19 and After COVID -19 periods, please see the supporting information (**S1 Methods**).

### Statistical analysis

Comparisons between population proportions and effect sizes pre-intervention and during intervention were expressed as odds ratios. NMR comparisons were illustrated by percentage change relative to pre-intervention and their confidence intervals obtained using the method described by the American Census Bureau for multi-year percentage changes, while MRS comparisons (sensitivity analysis) used Wilcoxon signed rank tests calculated using the R software. NMR comparisons were further stratified by country to account for epidemiological, operational, and contextual differences, and by healthcare tiers to highlight changes in relative contribution of the different tiers in the overall incremental percentage change measure observed across the network. All confidence intervals were calculated using a 95% confidence level and resulting p-values less than 0.05 were considered significant.

## Results

### Paediatric TB cascade

**Fig 1:**
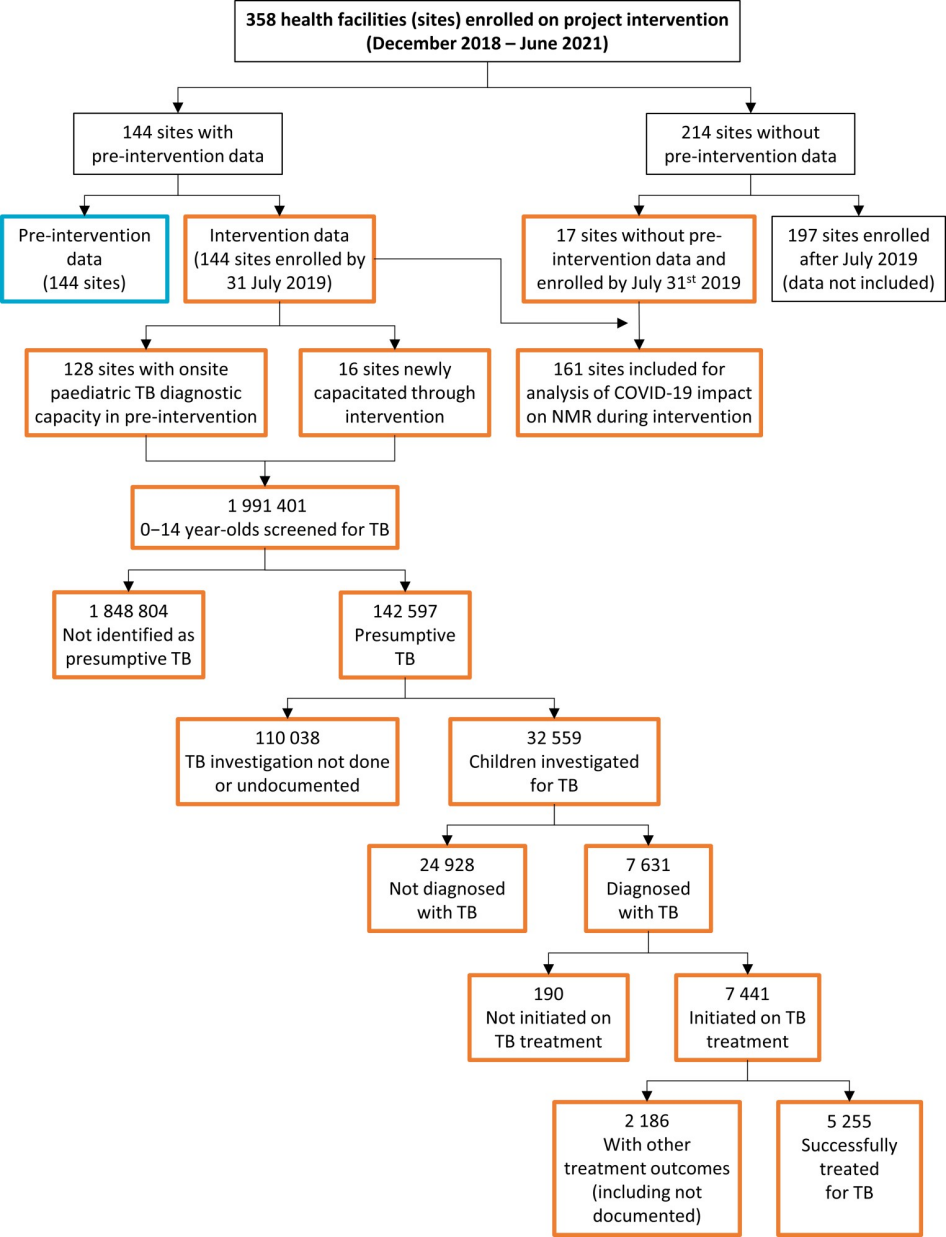
STROBE diagram. The diagram describes the distribution of health facilities among all sites enrolled on project intervention from December 2018 to June 2021, and their inclusion in the various analyses performed (pre- and during intervention comparison analyses, and analyses of impact of COVID-19 related restrictions on paediatric TB case detection within the intervention period). The diagram also describes the patient-level TB cascade, starting from 0–14 year old screened across all entry points, to those consequently successfully treated for TB as part of the intervention. Boxes are colour-coded to represent sites and data included in each of the analyses presented in this manuscript (pre-intervention = blue; during intervention = orange). NMR: network monthly rate; TB: tuberculosis.

Throughout the intervention period, 1 991 401 children 0−14 years of age were screened for TB across 144 CaP-TB sites with pre-intervention data (**Table 1 and Fig 1**). In total, 142 597 (7.2%) were identified as having presumptive TB, and of these, 5.4% (7 631) were diagnosed as having active TB. Of those diagnosed with active TB, 97.5% (7 441) were initiated on treatment. Among 5 742 children expected to have completed treatment by June 2021, 91.5% (5 225) achieved WHO-defined treatment success.

**Table 1:** Paediatric TB cascade of care, by entry point, during intervention.

Variable	Facility-based health care entry point						HCI	Total
	OPD	IPD	MCH	Nutrition	HIV Clinic	Undocumented		
**Number screened, n**	829 715	94 810	946 718	20 225	46 035	18 080	35 818	1 991 401
Entry point contribution to numbers screened	42%	5%	48%	1%	2%	1%	2%	100%
Proportion of presumptive TB cases* identified, (n/d)	8.8% (72 971/829 715)	18.5% (17 569/94 810)	4.4% (41 999/946 718)	11.9% (2 412/20 225)	6.4% (2 948/46 035)	9.1% (1 645/18 080)	8.5% (3 053/35 818)	7.2% (142 597/1 991 401)
Proportion of presumptive TB cases* investigated, (n/d)	24.7% (17 992/72 971)	29.5% (5 182/17 569)	5.4% (2 258/41 999)	14.0% (337/2 412)	93.0% (2 743/2 948)	60.4% (994/1 645)	100% (3 053/3 053)	22.8% (32 559/142 597)
**Number diagnosed with TB, n**	4 461	1 097	329	158	428	365	793	7 631
Entry point contribution to all TB cases diagnosed	58%	14%	4%	2%	6%	5%	10%	100%
Number needed to screen to detect one TB case, n (NNS)	186	86	2 878	128	108	50	45	261
**Number initiated on TB treatment, n**	4 319	1 077	323	155	425	362	780	7 441
**TB treatment initiated among diagnosed TB cases (n/d)**	96.8% (4 319/4 461)	98.2% (1 077/1 097)	98.2% (323/329)	98.1% (155/158)	99.3% (425/428)	99.2% (362/365)	98.4% (780/793)	97.5% (7 441/7 631)

Primary outcomes are those in bold. Abbreviations used correspond to: n/d, numerator/denominator; HCI, household contact investigations; IPD, inpatient department; MCH, maternal and child health; NNS, Number needed to screen; OPD, outpatient department

* Individuals screened for TB and presenting with at least one TB sign/symptom of chronic duration were defined as presumptive TB cases.

Most screening occurred in MCH and OPD services, which together contributed to 90% of the 1.99 million screened. Among the 7 631 children diagnosed with TB through project interventions, 6 838 (89.6%) were identified through facility-based intensified case finding (ICF), while 793 (10.3%) were identified through HCI. Among children diagnosed with TB through facility-based ICF, the majority was identified in OPD (4 461/6 838; 65.2%) and IPD (1 097/6 838; 16.0%), followed by HIV (428/6 838; 6.3%), MCH (329/6 838; 4.8%) and NUT (158/6 838; 2.3%). While the relative contribution to TB case finding by OPD was consistently the highest across all countries, some variability in relative contribution of other entry points was observed, notably in HIV (range 2–30%) and MCH (range 0–14%) services. The characteristics of entry points and the type of health services provided varied slightly across countries, especially for MCH and NUT entry points, and are summarized in S1 Table. In countries where unwell children with signs and symptoms of illness were attended in MCH (**S1 Table**), the relative contribution by MCH ranged from 12.5−13.5%, compared with 0−3.5% in countries where MCH services were restricted to routine care (e.g. routine immunisation and growth control; **S2 Table**).

The NNS was lowest in IPD (86) and HIV clinics (108), followed by NUT (128), OPD (186) and, MCH (2 878). NNS for HCI was 45. The proportion of children initiated on TB treatment was high irrespective of the entry point that represented the first point of contact with health services and ranged between 96.8 and 99.3%.

### Comparison of outcomes pre-intervention and during intervention

**Fig 2:**
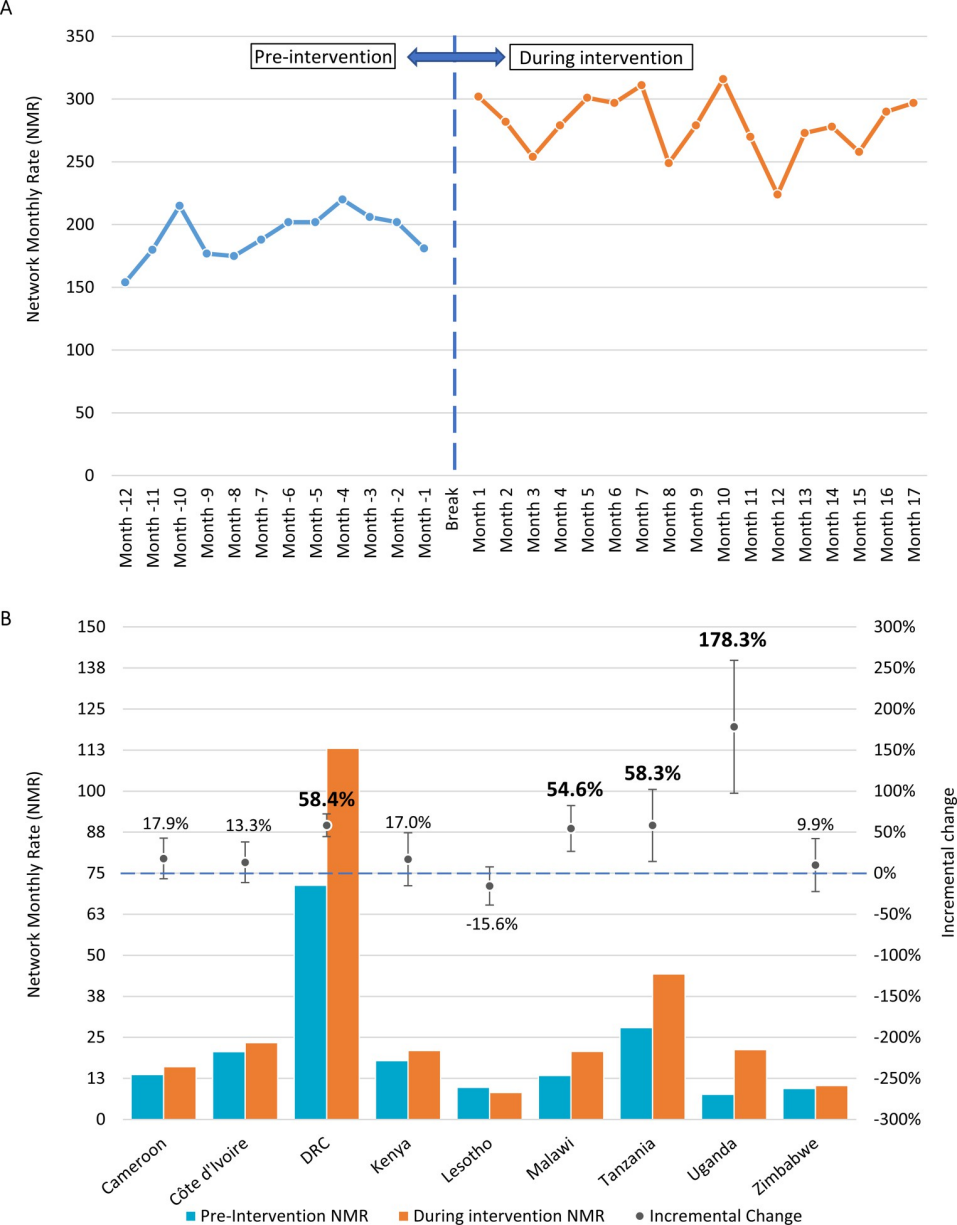
Pre-/during intervention comparison of the rate of 0–14 years old paediatric TB cases detected monthly across the network of 144 sites. (A) Monthly time series across all countries combined illustrating the change in trend of NMR before and during intervention periods. The period before intervention corresponds to a complete 12-month period ending at least six months before the date of retrospective data extraction. This period varied per country, but overall was between March 2017 and August 2018. The break period corresponds to the period where intervention data were incomplete for at least some of the sites for which pre-intervention data were collected in a given country, and Month 1 corresponds to the first month where all such sites were routinely implementing the intervention. As such, the intervention period displayed covers a 17-month period from April 2019 to June 2021, varying per country; (B) NMRs aggregated per country, before and during the intervention. Dots on the secondary y-axis represent the incremental percentage change (error bars representing the 95% confidence intervals) observed during intervention as compared with the monthly rate observed in the same sites during the pre-intervention period. Percentage changes in bold are statistically significant. DRC, Democratic Republic of the Congo; NMR, network monthly rate; TB, tuberculosis.

The NMR of children aged 0–14 years diagnosed with TB increased from 191.8 ± 18.4 pre-intervention to 280.0 ± 23.4 during the first 17 months of intervention, resulting in a statistically significant 46.0% increase (95% CI 36.2–55.8%, p<0.0001). The age disaggregated analysis showed that the NMR for children 0–4 and 5–14 years old diagnosed with TB increased by 53.4% (95% CI 35.2–71.9%, p<0.0001) and 39.9% (95% CI 27.6–52.2%, p<0.0001), respectively (**Table 2**). The monthly variations in the NMR pre- and during intervention is shown in **Fig 2A**. Statistically significant improvements were also noted when restricting the analysis only to the 128 facilities that already had on-site paediatric TB diagnostic capacity during pre-intervention. The NMR across these 128 sites increased by 41.5% (95% CI 31.7–51.4%, p<0.0001), from 191.8 ± 18.4 pre-intervention to 271.5 ± 24.9 during intervention (**S3 Table**). Consistent results were obtained when the sensitivity analysis, accounting for the entire duration of intervention for each site individually, was performed. The MRS had a significant median shift of 0.41 children aged 0–14 years diagnosed monthly per site (95% CI 0.25–0.63; p<0.0001), representing a 69.7% increase from 0.58 (IQR: 0.17–1.58) pre-intervention to 0.99 (IQR 0.39–2.46) during intervention (**S4 Table**).

**Table 2:** Network monthly rates (NMR) in paediatric TB case detection pre-intervention and during intervention.

	Pre-intervention (n = 144)*	During intervention (n = 144)*	Incremental change in % (95% CI)	p-value
**Number of months evaluated per site (mean ± SD)**	12.0 ± 0.0	17.0 ± 0.0	NA	NA
**Number of cases diagnosed with active TB**	2 302	4 760	NA	NA
**NMR 0–14 years old, mean (±SD)**	191.8 ± 18.4	280.0 ± 23.4	+46.0% (36.2–55.8%)	p<0.0001
**NMR 0–4 years old, mean (±SD)**	85.9 ± 16.1	131.8 ± 15.8	+53.4% (35.0–71.9%)	p<0.0001
**NMR 5–14 years old, mean (±SD)**	105.9 ± 12.3	148.2 ± 18.4	+39.9% (27.6–52.2%)	p<0.0001

Abbreviations used: CI, confidence interval; IQR, inter-quartile range, MRS, monthly rate per site; NA, not applicable; NMR< network monthly rate; SD, standard deviation

* n corresponds to the number sites, where 16 of the 144 sites sampled were newly capacitated in paediatric TB diagnosis through CaP-TB intervention (included in this comparison).

**Fig 3:**
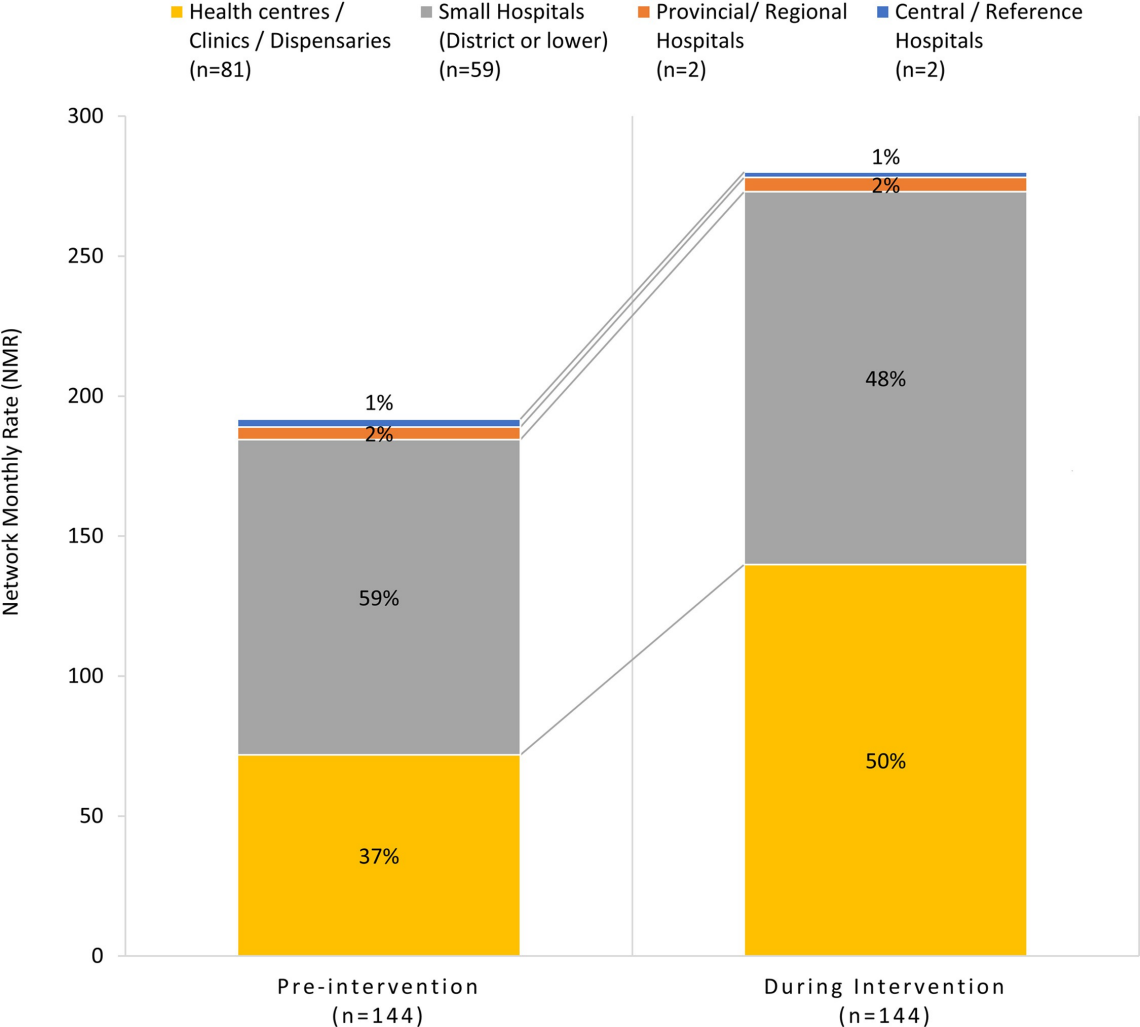
Changes observed in NMR of 0–14 year-old paediatric TB case detection disaggregated per healthcare tier categories pre-intervention and during intervention across the network of 144 sites. Healthcare tiers were grouped into similar service level categories to allow standardisation between countries. Categories were health centres, clinics, and dispensaries representing the lower tier, followed by all hospitals of district level or lower grouped as small hospitals, then provincial or regional hospitals, and finally central and reference hospitals. The height of the stacked bars represents the average monthly rate of paediatric TB case detection across the network before and during the intervention, whereas the relative contributions of each healthcare tier category is represented by percentage points (%) within each stacked bar to distinctively illustrate changes in paediatric TB diagnosis capacity within each tier of the network. NMR: network monthly rate; TB: tuberculosis.

Variability in the NMR was observed between countries, ranging from -15.6% to +178.3% (**Fig 2B**). Relative change in NMR was statistically significant in four countries, ranging from 54.6% (95% CI 26.7–82.5%, p<0.0001) in Malawi to 178.3% (95% CI 97.4–259.1%, p<0.0001) in Uganda. An increase in the number of children detected with TB was observed in four of the remaining five countries, although their relative changes in NMR were not statistically significant. A statistically significant 94.7% (95% CI 76.9–112.6%, p<0.0001) increase in the NMR was observed in lower-tier facilities (health centres/clinics/dispensaries), from 71.8 ± 9.1 pre-intervention to 139.9 ± 16.9 during intervention, while no statistically significant change was observed in intermediate and higher tiers (**S5 Table**). The relative contribution of lower-tier facilities to total cases detected improved from 37% (71.8/191.8) pre-intervention to 50% (139.9/280.2) during intervention (**Fig 3 and S5 Table**).

The proportions of children with bacteriologically confirmed TB (25.5% vs 15.2%, OR 1.9, 95% CI 1.7−2.2, p<0.0001) and the proportion diagnosed with pulmonary TB (75.6% vs 71.2%, OR 1.3, 95% CI 1.1−1.4, p<0.0001) were significantly higher during intervention compared to pre-intervention (**Table 3**). Although only a modest increase was observed in the proportion of TB cases initiated on treatment (97.5% vs 96.4%, OR 1.5, 95% CI 1.1−1.9, p = 0.004), treatment success rate improved to a greater extent (91.5% vs 78.9%, OR 2.9, 95% CI 2.5−3.3, p<0.0001).

**Table 3:** Paediatric TB cascade of care pre-intervention and during intervention.

	Pre-intervention	During intervention	Odds Ratio [95% CI]	p-value
**Number screened, n**	NA	1 991 401	–	–
**Proportion of presumptive TB cases identified, % (n/d)**	NA	7.4 (142 597 / 1 991 401)	–	–
**Proportion of presumptive TB cases investigated, % (n/d)**	NA	22.8 (32 559 / 142 597)	–	–
**Number diagnosed with TB, n (% among investigated)**	2 302 (NA)	7 631 (23.4)	–	–
Proportion bacteriologically confirmed, % (n/d)	15.2 (350 / 2 302)	25.5 (1 949 / 7 631)	1.9 [1.7–2.2]	<0.0001
Proportion diagnosed with pulmonary TB, % (n/d)	71.2 (1 639 / 2 302)	75.6 (5 770 / 7 631)	1.3 [1.1–1.4]	<0.0001
**Proportion initiated on TB treatment, % (n/d)**	96.4 (2 219 / 2 302)	97.5 (7 441 / 7 631)	1.5 [1.1–1.9]	0.0044
**Proportion successfully treated (among those with an expected treatment outcome at the time period analysed), % (n/d)**	78.9 (1 750 / 2 219)	91.5 (5 255 / 5 742)	2.9 [2.5–3.3]	<0.0001

Primary outcomes are those in bold. Abbreviations used: CI, confidence interval; n/d, numerator/denominator

### Impact of COVID-19

In order to assess whether COVID-19 pandemic related restrictions might have affected the impact of project interventions, we first compared the NMR pre-intervention versus during intervention, but limiting the data included in the intervention period to the Before-COVID period (**S1 File** and **S6 Table**). The relative increase in NMR ranged from 14.9% to 252.2% across countries, being statistically significant in five out of nine. Secondly, we compared the number of children diagnosed with TB monthly in distinct sub-periods of the intervention phase (Before COVID-19 period, COVID-19 period, and After COVID-19 period) (**S1 Fig**). In the Before COVID-19 period, the number of children diagnosed with TB detected monthly decreased (**S1A Fig**), with a statistically significant reduction in the NMR, from 288.3 ± 32.0 in the Before COVID-19 period, to 223.6 ± 18.2 in the COVID-19 period (22.5% reduction, 95% CI 30.3–14.6%, p<0.0001. The NMR in the After COVID-19 period was similar to the one observed in the Before COVID-19 period (290.0 ± 29.2; **S1B Fig**). A similar trend was observed when analysing the number of clinic attendees (0–14 years old) during the same three periods. The monthly average of clinic attendees decreased by 32%, from 150 600 during the Before COVID-19 period to 102 441 in the COVID-19 period (**S1A Fig**). While the absolute number of clinic attendees screened for TB also experienced a similar drop of 35% at the onset of the COVID-19 period (from 89 270 in March 2020 to 57 263 in April 2020), the proportion of screened among attendees decreased by only six percentage points (from 61% (89 270/147 204) in March 2020 to 55% (57 263/104 587) in April 2020). The number and proportion of attendees screened for TB progressively recovered during the COVID-19 period, to reach 80 600/106 710 (76%) in August 2020. While observing a partial recovery in the number of 0–14 years old accessing healthcare services in the After Covid-19 period (average number of attendees during this period was 112 175), levels did not reach those observed in the Before COVID-19 period. However, by the onset of the After COVID-19 period, the number of attendees receiving TB screening was comparable to that observed at the end of the Before COVID-19 period (86 556 in September 2020 vs 89 270 in March 2020) and this number varied minimally thereafter, with an average of 90 497 attendees screened for TB across the After COVID-19 period (**S1A Fig**). As a result, the number of children diagnosed with TB increased again reaching an average of 290 new TB cases detected per month in the After Covid-19 (compared to 288 in the Before Covid-19), despite a 26% decrease in number of clinic attendance (112 175 average number of clinic attendees in the After Covid-19 period vs 150 600 in the Before Covid-19 period). The impact of COVID-19 on paediatric TB case finding during the COVID-19 period was observed across the majority of project countries, with relative changes compared to the Before COVID-19 period ranging from -34.4% to -61.3% and a statistically significant decrease in the NMR in five of the nine project countries (**S1C Fig**). In addition, the NMR values in the After COVID-19 period vary considerably among countries, reflecting different relative capacity to promptly resume project activities and service delivery after the disruptions caused by the pandemic.

## Discussion

The CaP-TB multi-pronged intervention significantly increased the number of children and young adolescents <15 years diagnosed with TB. The effect was observed both for sites that did not notify paediatric TB cases during pre-intervention and for those already notifying paediatric TB cases during pre-intervention. This suggests that such interventions can not only build new capacity but can also strengthen existing capacity. In the majority of project countries, the increases in case finding were relevant from a public health viewpoint, especially as case detection accounts for the largest gap in the paediatric TB cascade of care. The interventions led to an increase in the number of children diagnosed with TB in eight out of the nine project countries and in four of them the increase observed was statistically significant. This study, which has a large sample size and is based on prospectively collected data, strengthens the evidence previously generated by single-country studies [12–15] and demonstrates that implementation of multi-pronged interventions to improve paediatric TB case detection is feasible and effective in routine clinical settings across a variety of geographical settings, although effect size may be variable depending on local context.

Several potential factors may explain why five of the nine countries did not observe a significant difference in paediatric TB case finding during the intervention. In two of these countries in West Africa and in Lesotho, our data suggest that HCWs were more prominently relying on bacteriologically confirmed diagnosis during the pre-interventions and this trend continued during most of the intervention period, as demonstrated by high proportions of children with bacteriological confirmation among those diagnosed with TB (**S7 Table**). Given difficulties in sample collection, especially among young children, and low sensitivity of currently available TB diagnostic assays for detection of TB in children, it may be that this high reliance on bacteriological confirmation resulted in missed cases. In Kenya and Zimbabwe, external factors such as economic crises as well as recurrent and widespread HCW strikes occurred during project implementation and likely impacted the effect of the CaP-TB programme [30, 31]. In addition, in Kenya the availability of integrated TB services for children and adolescents at the lower level of the health care system, was more prominently included under standard of care compared to other project countries [32]. This might have mitigated the effect of project interventions compared to pre-intervention standard of care.

The COVID-19 pandemic and related restrictions affected project interventions and patient access to health facilities. Project sites reported transport and movement restrictions, as well as hesitancy from both HCW and patients to provide and attend health services due to fear of contact with COVID-19 infected patients. Community-based contact investigation activities were heavily affected, with many countries temporarily suspending activities. This resulted in a reduction in the number of children and young adolescents diagnosed with TB. Our data suggests that this was mainly driven by the decrease in the number of individuals screened for TB which was primarily due to a reduction in clinic attendance, while the capacity to deliver TB screening was only marginally affected as confirmed by the moderate and time-limited decrease observed in TB screening coverage. The decreased impact on improved case finding was observed across all project countries, although to different extents. In three out of the five countries where no statistically significant effect of project interventions on paediatric TB case finding was observed (Kenya, Lesotho, and Zimbabwe), the network monthly rate of paediatric TB case detection remained low during the After COVID-19 period as compared to the Before COVID-19 period, suggesting a slower recovery from the disruptions caused by COVID-19. While COVID-19-related factors are not the sole cause for the mild impact of project interventions in certain countries, they contributed to the decreased paediatric TB diagnoses observed.

Through implementation of sample collection procedures and the support provided to access Xpert^®^ MTB/RIF or MTB/RIF Ultra testing, the CaP-TB intervention contributed to a statistically significant improvement in the proportion of children with bacteriologically confirmed TB.

Only 22.8% of presumptive TB cases were further investigated for TB during the intervention. This observed gap may be over-estimated because the project data collection system did not record how many children were prescribed a broad-spectrum antibiotic and requested to come back for a follow-up visit, or how many had an alternative diagnosis. However, other studies have documented similar gaps in the paediatric TB case detection cascade, suggesting this may be driven by clinicians’ reluctance to consider TB as part of their differential diagnosis [14, 28, 33, 34]. Intense training, site monitoring and mentorship, as well as improved access to tools such as molecular testing and radiology, may help increasing adherence to diagnostic algorithms and proportion of children undergoing TB investigations among those identified as presumptive.

More children diagnosed with TB were linked to care and initiated on treatment during intervention (97.5%) compared with pre-intervention (96.4%), and the number achieving treatment success considerably improved (91.5% versus 78.9%). This may be due to a combination of factors including more timely detection of TB due to the active case finding approaches implemented by the CaP-TB project, increased attention to regular follow-up of children while on treatment, increased data monitoring, or wider availability of child-friendly dispersible TB treatment during the intervention period. Treatment success rates achieved during the intervention are within the range reported globally by WHO [35], and by published retrospective studies [36–40], indicating that it is feasible to achieve these rates beyond the context of a well-supported project.

The age-disaggregated analysis of the NMR showed a greater increase in the 0–4 years age band compared to the 5–14 years. This represents an important achievement, as the TB detection and reporting gap is higher in this vulnerable population. In addition, the facility-level disaggregated analysis showed that the overall increase in the NMR of paediatric TB detection was driven by lower-tier facilities. This is consistent with findings from other studies conducted in sub-Saharan Africa showing significant gaps in paediatric TB care at the primary healthcare level [29, 34]; and strengthen the available evidence on the impact of interventions aimed at creating capacity to diagnose and manage paediatric TB in lower healthcare tiers on paediatric TB case finding [15, 16]. This highlights the importance of decentralising paediatric TB services, including through investing in frontline HCW capacity to diagnose and manage paediatric TB, and strengthening referral networks across tiers to enable linkage to care for children who need diagnostic investigations.

Most children diagnosed with TB were identified through integration of systematic paediatric TB screening in OPD and IPD, followed by HIV, NUT, and MCH. Among these facilities, the NNS was lowest in IPD, followed by HIV, NUT, OPD and MCH (although data on the relative contribution of nutrition services as limited by the low number of sites with separate NUT entry points). Of note, MCH only contributed a significant proportion of TB cases when this service was used to see children under five years of age who presented signs and symptoms of illness. National health programmes should map the patient-flow for ill children presenting to facilities and ensure that entry points that are designed to attend those children, systematically provide paediatric TB screening. HCI is also a key component of paediatric TB case finding interventions. HCI contributed to 10% of the total number of children diagnosed through the project and was characterised by a high NNS, supporting prioritisation of implementation and scale-up of this intervention [41, 42].

Our study had several limitations. As an observational, non-controlled before-and-after study, we could not control for time and other secular trends, including COVID-19, or differences in epidemiology of TB across participating countries. The data were collected over a relatively short period of time, however, no changes to paediatric TB case definition were made and no major new paediatric TB interventions were implemented during this time, partially mitigating the lack of control. The study relied entirely on routinely collected data for the pre-intervention period, while specific data collection tools and therefore data sources were introduced by the project, hence data sources pre-intervention and during intervention were different for several indicators. As the programme has been rolled out in a pragmatic manner and in collaboration with MoHs, purposive sampling was used to select sites, which may have led to bias in site selection.

Nevertheless, the study provides a reliable indication of potential achievements of adequately resourced and supported paediatric TB interventions. Based on the findings of this study, national programmes, donors, and technical agencies should ensure resources are available to implement comprehensive and multi-pronged approaches to paediatric TB case detection. In the context of limited resources, a phased approach could be considered to initially prioritise a core package of interventions, including: i) systematic paediatric TB screening in entry points designed to serve children presenting with signs and symptoms of illness (such as OPD and IPD) and/or in targeted services with lowest NNS, such as NUT and HIV; ii) systematic investigation of child contacts; iii) decentralisation of paediatric TB care into primary healthcare, including integration into health delivery packages for the management of illness in children under five years of age.

## Conclusion

The CaP-TB intervention although affected by the first COVID-19 wave, resulted in an increase in paediatric TB case finding under routine conditions in eight of nine project countries, including a statistically significant improvement in four of them. Despite important challenges in service delivery experienced during the COVID-19 period, including the time needed by countries to fully recover the functionality of TB services thereafter, this study represents, to the best of our knowledge, the first multi-country evaluation of a comprehensive and multi-pronged approach to improve TB detection in children and young adolescents, implemented under routine care conditions in the sub-Saharan African region. It is also the first multi-country study providing head-to-head comparison of case-finding yield achieved when ICF is implemented in different facility-level child health entry points. While acknowledging that the effect size of the multi-pronged approach may vary depending on settings and pre-existing standard of care, the evidence generated supports the recommendations on integrated and decentralized models of care included in the latest WHO guidelines on the management of TB in children and adolescents [20], providing additional practical insights on the key interventions to be prioritized to rapidly address the TB detection gap in this population.

## Supporting information

S1 Fig Comparison of NMR before, during and after the first COVID-19 wave across all intervention sites enrolled by July 2019. (A) Monthly time series across all countries combined illustrating the change in NMR trend “Before COVID-19”, “During COVID-19, and “After COVID-19”, as compared to changes in trend of number of individuals screened for TB among the 0–14 years old clinic attendees. Percentages represent the proportion of screened among attendees. The period During COVID-19 corresponding to the first wave (April 2020 to August 2020), and the period After COVID-19 corresponding to the period following the first wave (September 2020 to June 2021) are distinctively marked from the period Before COVID-19 onset (July 2019 to March 2020); (B) NMRs aggregated in all countries combined, Before, During, and After COVID-19. Dots on the secondary y-axis represent the relative percentage change observed after COVID-19 onset as compared with the NMR documented at the same sites Before COVID-19. Percentage changes in bold are statistically significant; (C) NMR disaggregated per country, Before, During, and After COVID-19. Dots on the secondary y-axis represent the relative percentage change observed through COVID-19 onset as compared with the NMR documented at the same sites Before COVID-19. Percentage changes in bold are statistically significant. DRC: Democratic Republic of the Congo; NMR: network monthly rate.
(TIF)

S1 Table Comparison of the various entry point characteristics present across the intervention countries. (PDF)

S2 Table Contribution of 0–14 years old TB cases detected in MCH entry point as compared to total detected among the 144 sites in countries where only routine services are available as compared to countries where under 5 years old sick children are attended by MCH. (PDF)

S3 Table Network monthly rates (NMR) in paediatric TB case detection pre-intervention and during intervention amongst the 128 facilities with pre-existing on-site paediatric TB diagnostic capacity during pre-intervention. (PDF)

S4 Table Monthly rate per site (MRS) in paediatric TB case detection pre-intervention and during intervention. The MRS comparison was disaggregated by sites previously capacitated in paediatric TB diagnosis.
(PDF)

S5 Table Comparison of the network monthly paediatric TB case detection rates disaggregated by healthcare tier before and during intervention. (PDF)

S6 Table Comparison of the network monthly paediatric TB case detection rates pre-intervention and during the pre-COVID-19 period of intervention, disaggregated by countries. (PDF)

S7 Table Comparison of the proportion of 0–14 years old TB cases detected that were bacteriologically confirmed amongst countries, pre-intervention and during intervention. (PDF)

S1 Checklist Questionnaire on inclusivity in global research. (PDF)

S2 Checklist STROBE checklist. (PDF)

S1 Methods (PDF)

S1 Protocol (PDF)

S1 Acknowledgments (PDF)

S1 Data (XLSX)
