# Supplementary figures and images for: Improving TB detection among children in routine clinical care through intensified case finding in facility-based child health entry points and decentralized management: A before-and-after study in Nine Sub-Saharan African Countries
Source: PLOS Glob Public Health. 2024 Feb 5;4(2):e0002865. doi: 10.1371/journal.pgph.0002865 (PMC10843113; doi:10.1371/journal.pgph.0002865)

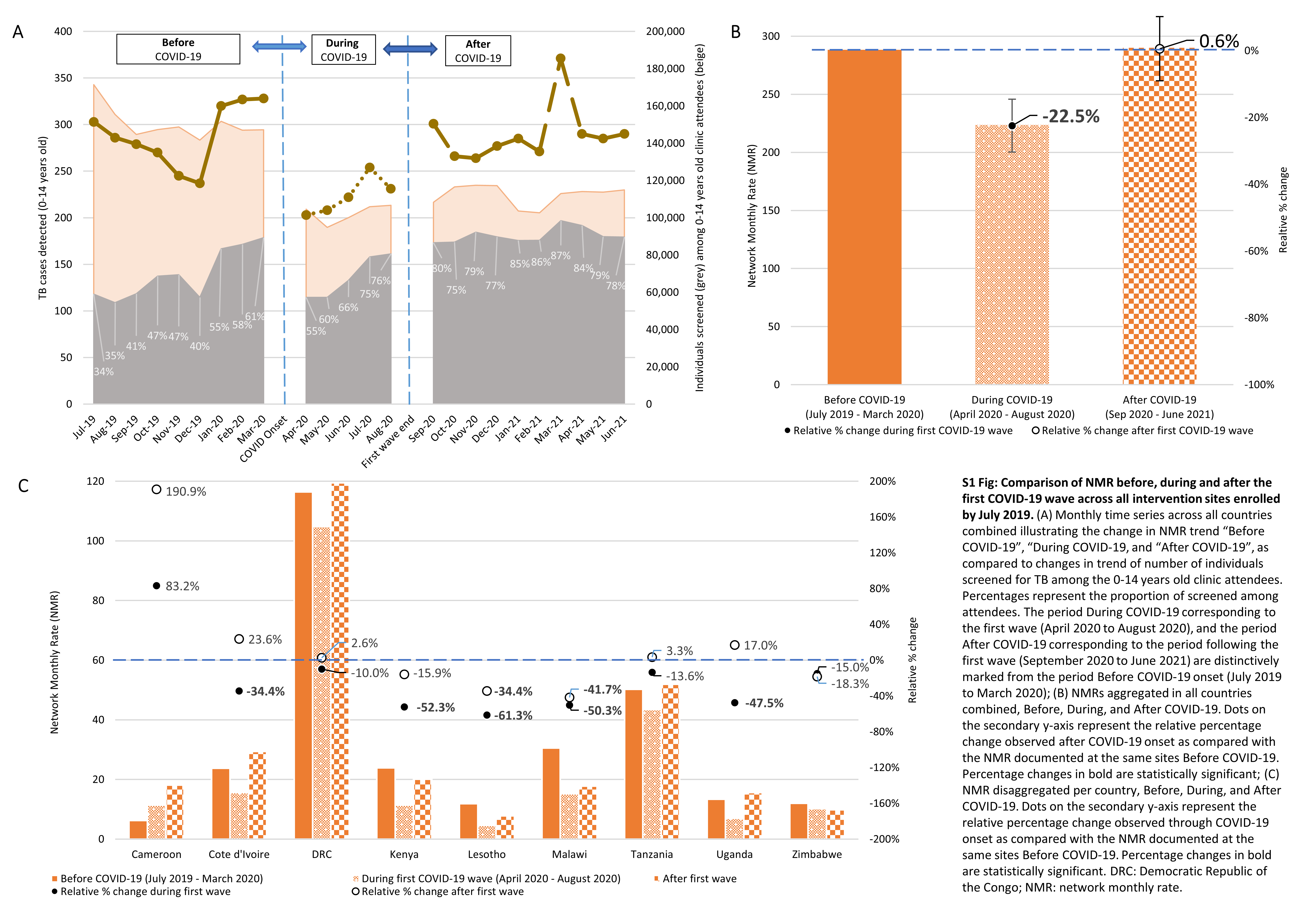

Supplement: S1 Fig — (A) Monthly time series across all countries combined illustrating the change in NMR trend “Before COVID-19”, “During COVID-19, and “After COVID-19”, as compared to changes in trend of number of individuals screened for TB among the 0–14 years old clinic attendees. Percentages represent the proportion of screened among attendees. The period During COVID-19 corresponding to the first wave (April 2020 to August 2020), and the period After COVID-19 corresponding to the period following the first wave (September 2020 to June 2021) are distinctively marked from the period Before COVID-19 onset (July 2019 to March 2020); (B) NMRs aggregated in all countries combined, Before, During, and After COVID-19. Dots on the secondary y-axis represent the relative percentage change observed after COVID-19 onset as compared with the NMR documented at the same sites Before COVID-19. Percentage changes in bold are statistically significant; (C) NMR disaggregated per country, Before, During, and After COVID-19. Dots on the secondary y-axis represent the relative percentage change observed through COVID-19 onset as compared with the NMR documented at the same sites Before COVID-19. Percentage changes in bold are statistically significant. DRC: Democratic Republic of the Congo; NMR: network monthly rate. (TIF) [file pgph.0002865.s001.tif]
