# Supplementary material for: Improving TB detection among children in routine clinical care through intensified case finding in facility-based child health entry points and decentralized management: A before-and-after study in Nine Sub-Saharan African Countries
Source: PLOS Glob Public Health. 2024 Feb 5;4(2):e0002865. doi: 10.1371/journal.pgph.0002865 (PMC10843113; doi:10.1371/journal.pgph.0002865)
Supplement: S1 Table — (PDF) [file pgph.0002865.s002.pdf]

S1 Table. Comparison of the various entry point characteristics present across the intervention countries

	OPD	IPD	MCH	NUT	HIV Clinic
**Cameroon**	I	I	R	I #	R
**Côte d'Ivoire**	I	NA	R	NA	NA
**DRC**	I	I	R	I #	R
**Kenya**	I	I	R*	I #	R
**Lesotho**	I	I	R	NA	R
**Malawi**	I	I	R	I	R
**Tanzania**	I	I	R*	NA	R
**Uganda**	I	I	R	I #	R
**Zimbabwe**	I	I	R**	I	R

I: Children presenting ill
R: Routine paediatric services for healthy children or children in HIV care
*: Sick children <5 years old are attended by MCH services at district hospital level only
**: Sick children <5 years old are attended by MCH services across all levels
#: Available as a separate service only at district hospital level or in few MoH selected facilities (<5 sites)
NA: Not available as the entry point did not exist as a standalone service in the intervention sites
