# Supplementary material for: Improving TB detection among children in routine clinical care through intensified case finding in facility-based child health entry points and decentralized management: A before-and-after study in Nine Sub-Saharan African Countries
Source: PLOS Glob Public Health. 2024 Feb 5;4(2):e0002865. doi: 10.1371/journal.pgph.0002865 (PMC10843113; doi:10.1371/journal.pgph.0002865)
Supplement: S2 Table — (PDF) [file pgph.0002865.s003.pdf]

S2 Table: Contribution of 0-14 years old TB cases detected in MCH entry point as compared to total detected among the 144 sites in countries where only routine services are available as compared to countries where under 5 years old sick children are attended by MCH.

	**Countries with only routine services in MCH (n=6)***	**Countries where sick children <5 years old are attended by MCH (n=3)****
**Number of children 0-14 years old detected in MCH entry points**	49	280
**Number of children 0-14 years old detected in total amongst all entry points**	5 491	2 140
**Contribution of MCH entry point to total TB case finding. % of total (n/d)**	0.9% (49/5491)	13.1% (280/2140)
**Range of the contribution of MCH to total TB case finding between countries**	0.0% – 3.5%	12.5% – 13.5%

This is Table S5 Legend: Abbreviations used: MCH, maternal and child health

* n corresponds to the number countries in which MCH entry offers routine paediatric services for healthy children or children in HIV care (Cameroon, Côte d'Ivoire, DRC, Lesotho, Malawi, Uganda).

** n corresponds to the number of countries in which sick children <5 years old are attended by MCH services, either at district hospital level only (Kenya and Tanzania), or across all sites (Zimbabwe).
