# Supplementary material for: Improving TB detection among children in routine clinical care through intensified case finding in facility-based child health entry points and decentralized management: A before-and-after study in Nine Sub-Saharan African Countries
Source: PLOS Glob Public Health. 2024 Feb 5;4(2):e0002865. doi: 10.1371/journal.pgph.0002865 (PMC10843113; doi:10.1371/journal.pgph.0002865)
Supplement: S3 Table — (PDF) [file pgph.0002865.s004.pdf]

S3 Table: Network monthly rates (NMR) in paediatric TB case detection pre-intervention and during intervention amongst the 128 facilities with pre-existing on-site paediatric TB diagnostic capacity during pre-intervention

	**Pre-intervention (n=128)***	**During intervention (n=128)***	**Incremental change in % (95% CI)**	**p-value**
**Number of months evaluated per site (mean ± SD)**	12.0 ± 0.0	17.0 ± 0.0	NA	NA
**Number of cases diagnosed with active TB**	2 302	4 615	NA	NA
**NMR 0-14 years old, mean (±SD)**	191.8 ± 18.4	271.5 ± 24.9	+41.5% (31.7–51.4%)	p<0.0001
**NMR 0-4 years old, mean (±SD)**	85.9 ± 16.1	128.5 ± 15.8	+49.5% (31.4–67.6%)	p<0.0001
**NMR 5-14 years old, mean (±SD)**	105.9 ± 12.3	143.6 ± 19.7	+35.6% (23.1–48.1%)	p<0.0001

This is Table S6 Legend: Abbreviations used: CI, confidence interval; IQR, inter-quartile range, MRS, monthly rate per site; NA, not applicable; NMR< network monthly rate; SD, standard deviation

* n corresponds to the number sites, where 16 of the 144 sites sampled were newly capacitated in paediatric TB diagnosis through CaP-TB intervention and were excluded in this comparison.
