# Supplementary material for: Improving TB detection among children in routine clinical care through intensified case finding in facility-based child health entry points and decentralized management: A before-and-after study in Nine Sub-Saharan African Countries
Source: PLOS Glob Public Health. 2024 Feb 5;4(2):e0002865. doi: 10.1371/journal.pgph.0002865 (PMC10843113; doi:10.1371/journal.pgph.0002865)
Supplement: S4 Table — The MRS comparison was disaggregated by sites previously capacitated in paediatric TB diagnosis. (PDF) [file pgph.0002865.s005.pdf]

S4 Table. Monthly rate per site (MRS) in paediatric TB case detection pre-intervention and during intervention.

	Number of sites*	Pre-intervention	During intervention	Incremental change in % (95% CI)	p-value
**Number of months evaluated per site (mean ± SD)**	**n=144**	12.0 ± 0.0	26.4 ± 2.9	NA	NA
	**n=128**	12.0 ± 0.0	26.6 ± 2.7		
**Number of cases diagnosed with active TB**	**n=144**	2 302	7 631	NA	NA
	**n=128**	2 302	7 420		
**MRS, median (IQR)**	**n=144**	0.58 (0.17–1.58)	0.99 (0.39–2.46)	**+0.41** (0.25–0.63)	$p<0.0001$
	**n=128**	0.58 (0.25–1.92)	1.12 (0.48–2.62)	**+0.42** (0.24–0.68)	$p<0.0001$

* The MRS comparison was disaggregated by sites previously capacitated in paediatric TB diagnosis and where n corresponds to the number sites included in the analysis, where 16 of the 144 sites sampled were newly capacitated in paediatric TB diagnosis through CaP-TB intervention, and were excluded in the analysis of 128 sites.
