# Supplementary material for: Improving TB detection among children in routine clinical care through intensified case finding in facility-based child health entry points and decentralized management: A before-and-after study in Nine Sub-Saharan African Countries
Source: PLOS Glob Public Health. 2024 Feb 5;4(2):e0002865. doi: 10.1371/journal.pgph.0002865 (PMC10843113; doi:10.1371/journal.pgph.0002865)
Supplement: S5 Table — (PDF) [file pgph.0002865.s006.pdf]

S5 Table. Comparison of the network monthly paediatric TB case detection rates disaggregated by healthcare tier before and during intervention

	**Number of sites** (relative %)	**Network Monthly Rates (mean ±SD)** (relative % within network)		**Incremental change** (95% CI)	**p-value**
		Pre-intervention (n=144)	Intervention (n=144)		
**Central / Reference Hospitals**	2 (1%)	2.8 ±1.7 (1%)	1.9 ±1.3 (1%)	-31.5% (-64.2%-1.2%)	NA
**Provincial / Regional Hospitals**	2 (1%)	4.6 ±2.2 (2%)	5.1 ±3.2 (2%)	10.4% (-34.1%-54.8%)	NA
**Small Hospitals (District or lower)**	59 (41%)	112.1 ±15.1 (58%)	133.1 ±21.2 (48%)	18.2% (5.6%-30.9%)	p=0.18
**Health centres / Clinics / Dispensaries**	81 (56%)	71.8 ±9.1 (37%)	139.9 ±16.9 (50%)	**94.7%** (76.9%-112.6%)	**p<0.0001**
**All sites combined**	144 (100%)	191.8 ±18.4 (100%)	280.0 ±23.4 (100%)	**46.0%** (36.2%-55.8%)	**p<0.0001**

NA, Not applicable
