# Supplementary material for: Improving TB detection among children in routine clinical care through intensified case finding in facility-based child health entry points and decentralized management: A before-and-after study in Nine Sub-Saharan African Countries
Source: PLOS Glob Public Health. 2024 Feb 5;4(2):e0002865. doi: 10.1371/journal.pgph.0002865 (PMC10843113; doi:10.1371/journal.pgph.0002865)
Supplement: S6 Table — (PDF) [file pgph.0002865.s007.pdf]

S6 Table. Comparison of the network monthly paediatric TB case detection rates pre-intervention and during the pre-COVID-19 period of intervention, disaggregated by countries.

	**Network Monthly Rates (mean ± SD)**		**Number of months of intervention data pre-COVID-19***	**Incremental change pre-COVID-19*** (95% CI)	**p-value**
	Pre-intervention (n=144)	Intervention pre-COVID-19* (n=144)			
**Cameroon**	13.7 ± 3.4	17.0 ± 8.0	2	24.4% (-56.6% — 107.4%)	p=0.37
**Côte d'Ivoire**	20.7 ± 4.5	23.9 ± 10.0	7	15.4% (-23.0% — 53.9%)	p=0.38
**DRC**	71.3 ± 8.5	112.0 ± 15.5	11	**57.0%** (40.4% — 73.6%)	**p<0.0001**
**Kenya**	17.9 ± 6.2	26.6 ± 10.4	10	**48.5%** (2.2% — 94.8%)	**p=0.03**
**Lesotho**	9.8 ± 2.4	12.0 ± 4.1	7	23.1% (-12.8% — 59.0%)	p=0.18
**Malawi**	13.4 ± 2.9	27.8 ± 4.1	12	**106.8%** (76.4% — 137.3%)	**p<0.0001**
**Tanzania**	28.0 ± 12.4	44.1 ± 12.3	7	**57.7%** (6.3% — 109.0%)	**p=0.02**
**Uganda**	7.7 ± 3.4	27.0 ± 6.1	6	**252.2%** (143.9% — 360.5%)	**p<0.0001**
**Zimbabwe**	9.4 ± 4.1	10.8 ± 5.9	11	14.9% (-31.6% — 61.4%)	p=0.53

*The period before intervention corresponds to a complete 12-month period ending at least six months before the date of retrospective data extraction. This period varied per country, but overall is between March 2017 and August 2018. As the enrolment of sites into intervention was progressive, the start of the period when all networked sites in a given country were enrolled varies per country, and therefore resulted in length of intervention period before the onset of COVID-19 for which the NMR could be measured varying per country and comprised between April 2019 and March 2020.
