# Supplementary material for: Improving TB detection among children in routine clinical care through intensified case finding in facility-based child health entry points and decentralized management: A before-and-after study in Nine Sub-Saharan African Countries
Source: PLOS Glob Public Health. 2024 Feb 5;4(2):e0002865. doi: 10.1371/journal.pgph.0002865 (PMC10843113; doi:10.1371/journal.pgph.0002865)
Supplement: S7 Table — (PDF) [file pgph.0002865.s008.pdf]

S7 Table: Comparison of the proportion of 0-14 years old TB cases detected that were bacteriologically confirmed amongst countries, pre-intervention and during intervention.

Countries	Pre-intervention (n=144)*	During intervention (n=144)*
**Cameroon,** % of bacteriologically confirmed cases 0-14 years old (n/d)	18.9% (31/164)	32.1% (105/327)
**Côte d'Ivoire,** % of bacteriologically confirmed cases 0-14 years old (n/d)	25.0% (62/248)	31.0% (189/609)
**DRC,** % of bacteriologically confirmed cases 0-14 years old (n/d)	12.9% (110/856)	35.9% (1 159/3 225)
**Kenya,** % of bacteriologically confirmed cases 0-14 years old (n/d)	17.2% (37/215)	8.7% (52/596)
**Lesotho,** % of bacteriologically confirmed cases 0-14 years old (n/d)	20.5% (24/117)	24.9% (51/205)
**Malawi,** % of bacteriologically confirmed cases 0-14 years old (n/d)	6.8% (11/161)	18.5% (117/631)
**Tanzania,** % of bacteriologically confirmed cases 0-14 years old (n/d)	12.5% (42/336)	10.6% (133/1 249)
**Uganda,** % of bacteriologically confirmed cases 0-14 years old (n/d)	15.2% (14/92)	14.4% (71/494)
**Zimbabwe,** % of bacteriologically confirmed cases 0-14 years old (n/d)	8.0% (9/113)	23.7% (70/295)
**All countries combined,** % of bacteriologically confirmed cases 0-14 years old (n/d)	14.8% (340/2 302)	25.5% (1 947/7 631)

This is Table S7 Legend: Abbreviations used: n, numerator; d, denominator.

* n corresponds to the number sites, where 16 of the 144 sites sampled were newly capacitated in paediatric TB diagnosis through CaP-TB intervention (included in this comparison).
