# Supplementary material for: Improving TB detection among children in routine clinical care through intensified case finding in facility-based child health entry points and decentralized management: A before-and-after study in Nine Sub-Saharan African Countries
Source: PLOS Glob Public Health. 2024 Feb 5;4(2):e0002865. doi: 10.1371/journal.pgph.0002865 (PMC10843113; doi:10.1371/journal.pgph.0002865)
Supplement: S1 Methods — (PDF) [file pgph.0002865.s011.pdf]

S1 Methods

Description of the intervention

The key and common elements of the interventions implemented across all countries are described below. However, it should be noted that approaches had to be adapted slightly depending on settings and context.

Paediatric TB training

To support these interventions, CaP-TB country teams organized Training of Trainers (ToT) workshops focused on paediatric TB management, in collaboration with MoH. The workshops were attended by both MoH and EGPAF staff. The personnel who received ToT training were then responsible for the training of frontline HCW in the project sites. Additionally, on-site training was implemented in most countries to ensure optimal HCW coverage in different entry points. In order to avoid interference with clinical work, on-site trainings were organized outside of peak attendance times. Job aids, such as desk cards describing paediatric TB screening and diagnostic algorithms were provided. Site mentorship and supervision visits were led by EGPAF staff jointly with facility-in-charge personnel whenever feasible. Site mentorship was usually provided 2, 4 and 6 weeks after the first training. If no major issues were identified during those three initial visits, follow-up visits occurred quarterly. District-level quarterly data review meetings were organized in collaboration with MoH staff. Separate training sessions were organized on sample collection procedures for HCWs identified as responsible for performance of the procedures, and laboratory personnel. Dedicated training sessions were provided to CHW or lay HCW engaged in contact investigation activities.

Chest X-ray services

Chest X-ray interpretation was supported by different means across the different countries: i) creation of informal working groups involving frontline HCW to allow for discussion and consultation with colleagues (all countries); ii) provision of subsidies for chest X-ray fees (Cameroon, DRC, Kenya, Lesotho, Tanzania); iii) establishment of a teleradiology network to support reading and interpretation of chest X-ray in children with presumptive TB (Kenya).

Laboratory-based TB diagnosis

The CaP-TB project supported the use of Xpert® MTB/RIF Ultra as the initial diagnostic test for TB diagnosis in children in the countries where national programmes had already included use of Xpert Ultra in national policies (Lesotho, Kenya, Uganda, Zimbabwe). In Cameroon, Côte d'Ivoire, DRC and Malawi, CaP TB piloted the use of Xpert Ultra at project sites (all sites or a subset of them depending on agreement reached with respective local authorities) upon assessing national TB programme interest in switching to the next generation Xpert® MTB/RIF cartridge and feasibility of scaling up its use beyond project scope and duration. The project did not specifically support introduction and use of Determine™ TB LAM Ag test in project countries but this assay was used for TB diagnosis in children living with HIV according to local policies and availability of the assay at facility level.

Contact investigation

In facility-based HCI, HCW requested index cases or caregivers of index cases to return to the facility with contacts (e.g. children living in the household of the index case) for evaluation. Contacts who did not return for evaluation were followed up through phone calls and/or CHW visits if possible. In community-based HCI, the TB focal person would debrief the TB index cases on the importance of checking the health status of

his/her house-hold contacts and request permission to visit the household. A CHW would visit the household of the index case to screen the child contacts. Symptomatic child contacts 0–14 years old were referred to facilities for diagnostic investigations. Asymptomatic children 0–4 years irrespective of HIV status and child contacts 5–14 years who were HIV positive were referred to a facility for confirmation of TB preventative treatment eligibility and initiation.

TB treatment

Treatment of TB cases was initiated according to MoH guidelines and used quality-assured dispersible fixed- dose combinations (FDCs) of rifampin (RIF)-isoniazid (INH)-pyrazinamide (PZA) with a separate tablet of ethambutol for

the intensive phase and dispersible fixed-dose tablets of rifampin-isoniazid for the continuation phase. It should be noted that site-level availability of the paediatric TB dispersible FDCs RIF 75mg/INH 50mg/PZA 150mg and RIF 75mg/INH 50mg varied among project countries. An assessment performed in six of the nine project countries during pre-intervention, showed that when dispersible FDCs were available in a given site, both formulations were generally introduced. However, the proportion of sites that introduced FDCs varied greatly between countries, ranging from 15% to 94%, with the majority of countries (six out of four) reporting paediatric dispersible FDCs being available only in 44% to 74% of the project sites. Dispersible tablets for treatment of TB were available in all CaP-TB sites during the intervention phase.

Human resources and financial support

Interventions were implemented by MoH staff assigned to facilities. The project supported the introduction of lay workers or CHW to perform paediatric TB screening in some countries and to perform community- based contact investigation interventions. When possible, civil society organizations (CSO) were involved in supporting community-based investigations. Food and transport reimbursements were provided to CHW. Phone air-time was provided to CHW and HCW to support patient follow-up. In Lesotho and Kenya, transport vouchers were provided to caregivers to support referral to facilities for TB diagnostic investigations (both for household child contacts identified as presumptive TB during community-based contact investigation and for children identified as presumptive TB through facility-based contact investigation and systematic TB screening at different child health entry points).

Methodology for analysis of the impact of COVID-19 related restrictions on paediatric TB case detection

For the post-hoc analysis assessing the impact of the COVID-19 pandemic on paediatric TB diagnosis during the intervention period, the NMR analysis was performed for including all intervention sites that had been enrolled in the project as of July 2019 (161 sites), irrespective of the availability of pre-intervention data. In this analysis, three fixed calendar periods were analysed across all countries. These periods were defined as the period prior to the first wave of the COVID-19 pandemic (July 2019- March 2020; referred to as the "Before COVID-19 period"); the period during the first wave of the COVID-19 pandemic (April 2020-Aug 2020; referred to as the "COVID-19 period"), and the period after the first wave of the COVID-19 pandemic (Sept 2020- June 2021; referred to as the "After COVID-19 period").[1] In addition, in order to measure the effect of project interventions prior to the COVID pandemic, we compared the NMR pre-intervention with the NMR during the pre-COVID intervention period across the 144 sites. The number of facility attendees was collected from facility records on monthly basis.

[1]Maeda JM, Nkengasong JN. The puzzle of the COVID-19 pandemic in Africa. *Science* 2021; **371**: 27–8.
