# Supplementary material for: Improving TB detection among children in routine clinical care through intensified case finding in facility-based child health entry points and decentralized management: A before-and-after study in Nine Sub-Saharan African Countries
Source: PLOS Glob Public Health. 2024 Feb 5;4(2):e0002865. doi: 10.1371/journal.pgph.0002865 (PMC10843113; doi:10.1371/journal.pgph.0002865)
Supplement: S1 Protocol — (PDF) [file pgph.0002865.s012.pdf]

Project title: **Evaluation of the Catalyzing Pediatric TB Innovation Project: A Pre- and Post-Implementation Assessment (TIPPI) across ten countries**

Protocol version 3. 3 dated 8th March 2021

Principal Investigator(s):

Martina Casenghi, PhD
Technical Director
Elizabeth Glaser Pediatric AIDS Foundation
WCC Center, 150, Route de Ferney, 1 Route des Morillons
PO Box 2100, Bureaux Jura 137 - 143, CH-1211 Geneva 2, Switzerland
mcasenghi@pedaids.org
+39 329 141 1374

Jean-François Lemaire, MSc
Diagnostics & Systems Lead
Elizabeth Glaser Pediatric AIDS Foundation
WCC Center, 150, Route de Ferney, 1 Route des Morillons
PO Box 2100, Bureaux Jura 137-143, CH-1211 Geneva 2,
Switzerland
Jlemaire@pedaids.org
+41 22 791 6457

Co-investigators:

Mikhael de Souza
Project Director
Elizabeth Glaser Pediatric AIDS Foundation
WCC Center, 150, Route de Ferney, 1 Route des Morillons
PO Box 2100, Bureaux Jura 137 - 143, CH-1211 Geneva 2, Switzerland
mdesouza@pedaids.org
+41 76 376 41 77

Shirin Kakayeva, MPP
Senior Strategic Information and Evaluation Officer
Elizabeth Glaser Pediatric AIDS Foundation
1140 Connecticut Ave., NW, Suite 200
Washington, DC, USA
skakayeva@pedaids.org
+1 (202) 280-1640

Rhoderick Machekano, PhD, MPH
Senior Biostatistician
Elizabeth Glaser Pediatric AIDS Foundation
1140 Connecticut Ave., NW, Suite 200
Washington, DC, USA
rmachekano@pedaids.org
+1 (202) 470-6654

Boris Tchounga, PhD
Associate Director of Public Health Evaluation – Cameroon
Elizabeth Glaser Pediatric AIDS Foundation

Patricia Fassinou Ekouévi, MD
Country Implementation Manager – Cote D'Ivoire
Elizabeth Glaser Pediatric AIDS Foundation

Vicky Ilunga, MD
Country Implementation Manager – DRC
Elizabeth Glaser Pediatric AIDS Foundation

Phelix Mboya, MD
Country Implementation Manager – Kenya
Elizabeth Glaser Pediatric AIDS Foundation

Samson Lanje, MD
Country Implementation Manager – Lesotho
Elizabeth Glaser Pediatric AIDS Foundation

Sathish Kumar, MD
Country Implementation Manager – India
Solidarity and Action Against The HIV Infection in India

Yusuf Bhamu, MD
Country Implementation Manager – Malawi
Elizabeth Glaser Pediatric AIDS Foundation

Leo Haule, MD
Country Technical Advisor for TB – Tanzania
Elizabeth Glaser Pediatric AIDS Foundation

Mary Namubiru, MDCountry Implementation Manager – Uganda
Elizabeth Glaser Pediatric AIDS Foundation

Tichaona Nyamundaya, MD
Country Implementation Manager – Zimbabwe

Elizabeth Glaser Pediatric AIDS Foundation

Project Locations:
Cameroon, Cote d'Ivoire, Democratic Republic of the Congo, Kenya, Lesotho, India, Malawi, Tanzania, Uganda, Zimbabwe

Proposed Project Dates:
EGPAF Project: October 1, 2017-September 30, 2021
Data collection activities: October 2017-September 2021

Table of Contents

ACRONYMS ... 7

I. Rationale & Background Information ... 8

II. Project Goals and Objectives ... 9

A. Goal ... 9

B. Objectives ... 10

1. Primary Objective ... 10

2. Secondary Objectives ... 10

III. Expected Outcomes of the Study ... 11

IV. Study Design ... 11

A. Methodology ... 11

1. Description of the CaP TB Project ... 11

2. Country-Level Activities ... 13

3. Duration of the Project ... 14

V. Description of Project Evaluation ... 14

A. Pre-intervention Evaluation- Baseline assessment ... 15

VI. Evaluation Design and Methods ... 16

A. Table 1. Indicator Outcomes and Data Sources ... 18

B. Table 2. Additional Indicator Outcomes and Data Sources ... 20

VII. Use of data generated through CaP TB program evaluation to perfom secondary analysis ... 21

A. Use of CaP TB Program evaluation data to inform cost-effectiveness analysis ... 21

B. Use of TIPPI data to assess the impact of COVID 19-related measures on accessibility and delivery of pediatric TB services ... 22

VIII. Data Gathering Activities ... 23

A. Table 3. Study Data Gathering Activities During Pre-intervention and CaP TB Intervention Phases ... 23

IX. Data Management and Statistical Analysis ... 24

A. Quality Assurance ... 24

X. Privacy and Confidentiality ... 25

XI. Ethical Considerations ... 27

A. IRB Review ... 27

B. Ethics Training ... 27

C. Conflict of Interest 27
D. Informed Consent 27
E. Risk and Benefits to Participants 28
F. Follow-Up 28
XII. Dissemination of Results and Publication Policy 28
XIII. Problems Anticipated 29
XIV. Project Management 29
XV. Annex A: CaP TB Information System 30
References 31

ACRONYMS

WHO-AFRO	WHO Africa Regional Office
BMU	Basic Management Unit
CaP TB	Catalyzing Pediatric TB Innovation
DOB	Date of Birth
DRC	Democratic Republic of the Congo
DR-TB	Drug-resistant Tuberculosis
DS-TB	Drug-sensitive Tuberculosis
EGPAF	Elizabeth Glaser Pediatric AIDS Foundation
EPTB	Extra pulmonary TB
GF-ERP	Global Fund Expert Review Panel
HCW	Health Care Worker
HF	Health Facility
HIV	Human Immunodeficiency Virus
HQ	Headquarters
ICF	Intensified Case Finding
IPT	Isoniazid Preventive Therapy
IQR	Interquartile Range
IRB	Institutional Review Board
M&E	Monitoring and Evaluation
MDRTB	Multidrug-resistant TB
MNCH	Maternal, Neonatal and Child Health
MOH	Ministry of Health
MOU	Memorandum of Understanding
MTB	Mycobacterium Tuberculosis
MTB-Rif	Mycobacterium Tuberculosis Rifampicin
NNS	Number Needed to Screen
NTP	National Tuberculosis Programs
PMTCT	Prevention of Mother to Child Transmission
PT	Preventive therapy
PTB	Pulmonary TB
QA	Quality Assurance
RIF	Rifampicin
SAATHII	Solidarity and Action against the HIV Infection in India
SEARO	WHO Southeast Asia Regional Office
SIE	Strategic Information and Evaluation
SOP	Standard Operating Procedure
TA	Technical Advisor
TAT	Turn Around Time
TB	Tuberculosis
Xpert	GeneXpert
WHO	World Health Organization

I. Rationale & Background Information

Tuberculosis (TB) in children is often missed or overlooked due to non-specific symptoms and difficulties in diagnosis. This has made it difficult to assess the actual magnitude of the childhood TB epidemic, which may be higher than currently estimated.[I] Due to this paucity of data, the extent of TB in children remains largely unknown.[II] In 2016, the World Health Organization (WHO) estimated that over one million children (<15 years) suffer from TB worldwide. With over 201,000 child deaths globally in 2016, including 52,000 among HIV/TB co-infected children, TB is a top ten cause of death in children. HIV co-infected children and the general population of children under five years of age (irrespective of HIV infection) are also at higher risk of pediatric TB.[III]

Significant gaps exist across the entire cascade of care for pediatric TB including prevention, screening, diagnosis and treatment for pediatric TB. Globally, only 39% of pediatric TB cases are notified to national TB programmes (NTPs), with the remaining children undiagnosed or unreported.[IV]. Those who are not diagnosed experience high case fatality rates of 50% or more.[V] [VI] A recent analysis of the cascade of care for pediatric TB in Kenya and Uganda demonstrated that only 20% of the estimated pediatric TB cases in the cohort completed TB treatment, with the largest shortfall being in diagnosing patients.[VII]

The heaviest burden of pediatric TB is in sub-Saharan Africa and South Asia. Africa accounts for about one-third of all pediatric TB cases,[VIII] with an incidence of 29-34/100,000, which is double of the global average.[IX] In HIV-endemic African countries, 40-60% of pediatric TB patients are also infected with HIV.[X,XI] At 23% of pediatric TB cases, sub-Saharan Africa disproportionately contributes to the global pediatric TB burden. Further, at 46% of the global pediatric TB mortality, sub-Saharan Africa disproportionately contributes to pediatric TB mortality.[XII] Sub-Saharan Africa leads in the contribution to TB under-diagnosis and under-reporting. Weak case finding, lack of and underuse of diagnostic tools, inadequate linkage to care, and lack of appropriate pediatric TB formulations result in poor clinical outcomes.[XIII,XIV]

With the estimated incidence of 2.8 million TB cases in 2017, India is the highest TB burden country in the world in terms of absolute numbers of incidence cases each year. Due to the heavy burden and large population, India accounts for the largest number of pediatric TB cases globally, with an estimated 255,000 incident pediatric TB cases in 2015.[XV] Pediatric TB makes up 6% (approx 0.1 million) of notified 1.8 million cases. However, the exact scope of the epidemic is unknown, due to inadequate data and surveillance systems. Additionally, nearly half of all TB patients are treated in the private sector, and many are not notified to the National TB Program.[XVI]

Models of care that include service integration and decentralization may improve clinical outcomes for children living with TB. Recently, critical progress has been achieved after years of neglect and underfunding. At the global policy level, the WHO post-2015 End TB Strategy,[XVII] the Stop TB Partnership Global Plan to End TB 2016-2020,[XVIII] and the Roadmap for Childhood TB have raised global awareness, developed a global policy framework and guidance to support national pediatric TB programmes,[XIX] and set a global target of providing effective TB treatment to 90% of key populations living with TB, including children, by 2020. The Roadmap for Childhood TB highlights the key priorities of developing integrated strategies for screening, diagnosis, and care; developing training materials for health workers; and collecting and reporting

better data on pediatric TB. Improved diagnostics such as the GeneXpert MTB-Rif system have also entered the market and may help improve diagnosis of pediatric TB.[XX]

Very recent data from several small single-country studies have demonstrated that integration of TB interventions into other healthcare entry points can improve outcomes for pediatric TB.[XXI] Growing evidence from other disease areas like HIV, including HIV and TB service integration, have shown that integration and decentralization results in good clinical outcomes.[XXII, XXIII] In the era of the Sustainable Development Goals, with a focus on universal health care, developing, documenting, and catalysing the scale-up of innovative models of care focused on integration is a timely and critical intervention. In addition, the HIV pediatric community recently released their research prioritization which included a call for evidence generation for "optimal prevention and clinical management of co-infections, particularly tuberculosis" which includes implementation strategies such as integration.[XXIV]

While policies, such as those noted above and in-country policies to use improved diagnostics (e.g. Xpert) and provide TB preventive therapy, have been strengthened, the proposed strategies are still poorly implemented. A recent analysis of pediatric TB programmes in 12 African countries shows that about 40% of countries still rely on sputum smear microscopy as the primary diagnostic tool despite the wide knowledge of challenges related to sputum production in children under-five and the WHO recommendation (issued in 2018) to use Xpert MTB/RIF as the initial diagnostic test for diagnosis of childhood TB.[XXV] In addition, 64% noted very limited use of Isoniazid preventive therapy (IPT) in HIV-infected children.[XXVI] Globally only 7% of eligible children were started on preventative therapy in 2015.[XXVII]

General underfunding of TB programs[XXVIII] and lack of dedicated resources for pediatric TB [XXIX] has historically hindered effective implementation and led to the gap described above. Strategic allocation of resources towards most impactful intervention is critical in such context. Costing and cost-effectiveness data could help informing national programs planning and budgeting but evidence currently available for childhood TB remains limited.
Given the critical and urgent need to improve models of care for pediatric TB that include strengthened case finding and use of innovative diagnostics and drugs that may offer improved outcomes for children with TB infection or disease, The Elizabeth Glaser Pediatric AIDS Foundation (EGPAF) is implementing the Unitaid-funded Catalyzing Pediatric TB Innovation (CaP TB) project. CaP TB will develop and implement innovative models of care and accelerate the broad introduction of WHO-recommended and quality-assured drugs and diagnostics that will help bring care closer to children living with and at risk for TB and close the pediatric TB gap.

This protocol describes the program evaluation that will help to document the outcomes of the CaP TB project.

II. Project Goals and Objectives

A. Goal

To evaluate the effectiveness of the CaP TB project across ten countries on key service delivery and clinical outcomes and compare these outcomes to those obtained at baseline.

B. Objectives

1. Primary Objective

1. To evaluate the below TB service indicators and clinical outcomes for children 0-14 in facilities implementing the CaP TB project:
2. To evaluate the below TB service indicators and clinical outcomes for children 0-14 in facilities implementing the CaP TB project:
    a. Number and proportion of children screened for TB among clinic attendees
    b. Number and proportion of presumptive TB cases identified among children screened for TB
    c. Number and proportion of presumptive TB cases referred for lab-based TB diagnosis
    d. Number and proportion of pediatric presumptive TB cases who are tested with Xpert
    e. Number and proportion of pediatric TB cases diagnosed with active TB among presumptive pediatric TB cases.

    f. Number and proportion of presumptive TB cases identified among children screened for TB
    g. Time between when a child is identified as a presumptive TB case and when the child is diagnosed with TB
    h. Number and proportion of pediatric TB cases started on DS-TB treatment
    i. Time between when a child is identified as a presumptive TB case and when the child is initiated on TB treatment
    j. Number and proportion of pediatric DS-TB cases or cases treated with first-line TB treatment who achieve treatment success
    k. Number and proportion of all TB index cases for whom successful contact tracing has been done
    l. Number and proportion of pediatric household contacts who are negative to TB screening
    m. Number and proportion of screened negative pediatric contacts eligible for preventive therapy
    n. Number and proportion of pediatric patients started on preventive therapy of those who are eligible for preventive therapy
    o. Number and proportion of pediatric patients on preventive treatment who completed therapy

2. Secondary Objectives

a. To determine the effect of the CaP TB project on the above TB service indicators and clinical outcomes compared to baseline (standard of care) in a subset of project sites.

b. Utilize the evidence generated from this program evaluation to support strategic recommendations to improve pediatric TB care.

c. Utilize clinical aggregated data generated by this study to inform cost-effectiveness analysis of pediatric TB interventions implemented by CaP TB project that can contribute informing childhood TB planning and strategic allocation of financial resources

d. Utilize evidence generated from this program to assess the impact of measures enforced to control the COVID -19 pandemic on accessibility and availability of pediatric TB services

III. Expected Outcomes of the Study

The main expected outcome of CaP TB project is to reduce morbidity and mortality associated with pediatric TB[xxx] by reducing the pediatric TB diagnostic and treatment gap, increasing coverage of quality assured TB treatments, as well as scale up of TB preventive therapy through innovative models of care for pediatric TB that focus on integration and decentralization. We expect that this program evaluation will document a positive impact of the CaP TB model, with estimated two-fold increase in case detection and four-fold increase in pediatric patients started on TB preventive therapy based on project target estimates. The evaluation of the CaP TB project will allow for dissemination of the results and further uptake of proven models by other countries.

IV. Study Design

We are planning a pre-/post-project (quasi experimental) intervention evaluation. The population will include all pediatric patients (0-14 years of age) who present to a CaP TB project site in any of the 10 countries and receive TB services (including screening, diagnosis and treatment for active TB, and provision of TB preventive therapy).

A. Methodology

1. Description of the CaP TB Project

The CaP TB project is a 4-year (October 1, 2017-September 30, 2021) Unitaid-funded project implemented by EGPAF (in sub-Saharan Africa) and SAATHII (in India) to improve pediatric TB outcomes by introducing models of care and strengthening access to WHO-recommended drugs and diagnostics. EGPAF and SAATHII, through the CaP TB project will develop, implement, and document innovative models of pediatric TB care focusing on integration, decentralization, and community-based care. Integration refers to supporting TB activities, such as screening and TB sample collection for children, into non-TB health care services, such as general pediatric outpatient clinics. Decentralization refers to moving pediatric TB services from higher levels of health clinics to lower levels. Community-based care refers to care that is delivered outside the facility, often, but not exclusively, at household level. We hypothesize that such models of care will increase case detection and care delivery, thus improving key pediatric TB outcomes. CaP TB focuses on the regions with the largest contributions to pediatric TB: AFRO and SEARO. The CaP TB countries where the project and project evaluation will be carried out are: Cameroon, Cote

d'Ivoire, DRC, Kenya, Lesotho, India, Malawi, Tanzania, Uganda and Zimbabwe.

Innovative models of care will include integration of TB services into MNCH, HIV, nutrition, and other key pediatric health care entry points. CaP TB will also support improved intensified community case finding including through contact tracing of adult and pediatric index TB patients, optimized clinical-radiological diagnosis, increased uptake of molecular diagnostics, and where feasible, decentralized or community-based initiation of TB preventive therapy. CaP TB will not be altering treatment algorithms for TB preventive therapy or for those with active infection, but will be following national guidelines or MOH guidance to the project as to the type and duration of treatment provided.

a) Figure 1: Intensified Case Finding & Integration of Services

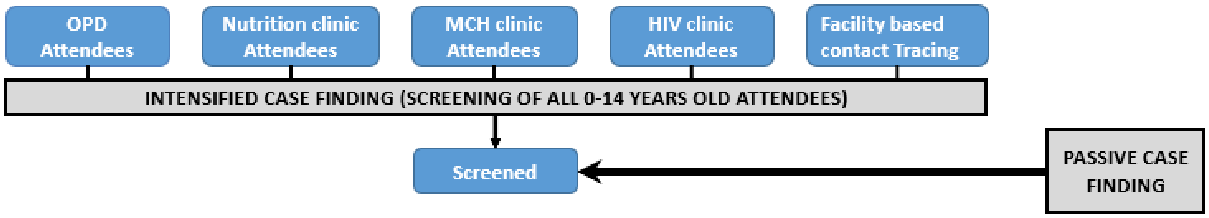

While most of the CaP TB project will be implemented in public facilities, in some EGPAF-supported countries, EGPAF has worked with the private sector, including faith-based clinics. EGPAF will also support select private health facilities that EGPAF currently supports to adopt CaP TB project interventions, including integration of care and improved models of care for case finding and uptake of TB preventive therapy. In India, SAATHII has focused much of its work supporting the private sector through public-private partnership models. These models have shown considerable promise to improve TB care in India and similar contexts and SAATHII has utilized this model to improve pediatric care in the private sector in India.[XXXI]

Initially, CaP TB will be implemented in approximately 15-20 purposefully selected and diverse sites per country for the first 2 years of the project (October 2017-October 2019) to refine approaches, determine costs, and inform a second phase of expanded implementation to prove scale-ability, avoid interventions becoming stuck in the pilot phase, and bridge to scale up. Data gathered will be used to demonstrate the effectiveness and feasibility of innovative interventions. During the last six months of this intensive monitoring phase, EGPAF will analyze the results and lessons learned from early implementation of these innovative models of care. Results will be discussed with key in-country and international stakeholders in order to help refine the models and plan for a period of expanded implementation.

EGPAF will then enter into a period of expanded implementation that will help to prove and catalyze scalability and bridge to transition. The number of sites chosen for the expanded implementation phase was selected to ensure sufficient strength of evidence to induce catalytic change nationally, be generalizable and build a good basis for eventual transition and scale up. Thus, within each country, EGPAF's site selection aimed to include: 1) Multiple types of sites including different health facility levels; 2) varying health entry points (e.g. nutrition centers, sites

with pediatric inpatient wards, etc.); 3) diverse geographic setting and ease of access to health facilities; and 4) different population types (including key populations such as refugee populations, fisher folk). Depending on the size of the country and the diversity of the contexts within each country, this resulted in a varying number of sites per country.

2. Country-Level Activities

EGPAF and SAATHII have strong expertise in implementing innovative service delivery models and will provide training, supportive supervision, job aids, and standard operating procedures (SOPs) to health care workers who are engaged in the care of pediatric patients at CaP TB facilities. The goal of these activities is to support the strengthened uptake of current recommendations on TB screening, diagnosis and treatment as well as allow for decentralization and integration of pediatric TB care. CaP TB will also provide training to strengthen completion of TB registers, and may develop other M&E tools to improve the documentation of pediatric TB. EGPAF and SAATHII will also assess and provide SOPs and infrastructure upgrades to improve infection control. EGPAF and SAATHII will support innovations to improve service delivery including creating facility-specific TB champions to monitor service quality, regular performance feedback to facilities, and work with civil society to identify areas to improve care.

Specific packages of activities will vary by country and will be determined based on (1) national policies and guidelines; (2) planned and existing activities supported through other efforts; and (3) country-specific pediatric TB burden and barriers and bottlenecks. For Country Submission- refer to country specifics.

In general, country-level activities will include:

- Decentralizing and integrating delivery of pediatric TB care (diagnosis, treatment (where feasible), and prevention) into MNCH, HIV, nutrition and other key pediatric entry points at the facility level;
- Supporting community-level contact tracing and screening of pediatric contacts for index patients with active TB and where feasible according to MOH guidance, initiation of preventative TB therapy at the community level;
- Documenting and generating evidence for innovative models of care through M&E;
- Strengthening data systems through support for improved use of current registers as well as introduction of new data tools where gaps exist (e.g. for tracking of TB preventive therapy completion in some countries);
- Supporting rapid uptake through trainings, site monitoring and mentorship and strengthening sample transport and referral systems of new diagnostics and medicines that are included in WHO guidelines and are either included in country guidelines or are in the process of being incorporated into national guidance, such as:
  - GeneXpert diagnostic assays (i.e Xpert MTB/RIF Ultra)[XXXII]
  - Quality-assured pediatric dispersible formulations of fixed dose isoniazid-rifampicin-pyrazinamide and rifampicin-isoniazid as well as ethambutol single formulation for the treatment of active TB disease;

- Quality-assured pediatric formulations of dispersible isoniazid single formulation and fixed dose rifampicin-isoniazid for preventive therapy[XXXIII].

- Supporting the introduction and use of e-/m-health technologies (i.e connectivity solutions, SMS-reminders) to strengthen performance of TB screening at household level, linkage to care and/or patient's treatment adherence strategies

Drugs and diagnostics used in CaP TB must have approval from a stringent regulatory agency or be recommended by the Global Fund ERP, the WHO prequalification program or WHO Global TB program and be registered by the country national regulatory authority or have an exemption for import and routine use in the country. Cepheid recommends that gastric aspirate (GA) samples tested on the Cepheid GeneXpert platform be centrifuged to improve yield. As some of our Xpert testing sites will not have access to the appropriate centrifuge and infection control equipment, we will use direct GA specimens without centrifugation for Xpert testing. Testing without a concentration step (achieved through centrifugation) may reduce the sensitivity of Xpert testing for this specific sample type. It will not affect specificity. As the sensitivity of GA even with a centrifugation step is estimated to be 83% according to a WHO meta-analysis, WHO guidelines[XXXIV] recommend that a negative result from a GA sample tested on Xpert not be used to definitively rule out TB. As the sensitivity from testing without a centrifugation step may be lower, we will stress in our trainings that a negative result from an Xpert test on GA does not rule out TB. This practice will be reviewed with the National TB Program before implementation.

3. Duration of the Project

Data collection of pre-intervention retrospective baseline data collection will take place in the months prior to or immediately after the start of CaP TB project interventions. Post-intervention data collection will take place once the facility is enrolled in CaP TB and will be on-going over the course of the project (through September 30, 2021). It is expected that most countries will begin enrolling pilot facilities by September 2018.

V. Description of Project Evaluation

The CaP TB model represents one potential way of improving the care of pediatric TB. However, it will be important to measure this model's effect on key service and clinical indicators related to pediatric TB in order to help guide future programming and resource prioritization. In order to describe the effect of the CaP TB model on these indicators, we plan a pre- and post-intervention evaluation in all project countries. We have chosen this pragmatic approach to allow us to compare the outcomes after we begin CaP TB implementation with the same indicators baseline (pre-implementation). This can help us define the effect of our specific project on the project evaluation indicators. As this is an evaluation of program implementation a randomized approach would not be feasible for both programmatic and financial reasons. We do recognize that secular trends may affect our outcomes, but we feel that the duration of the evaluation (over several years) and the large size and multi-country nature will help to mitigate that risk.

A. Pre-intervention Evaluation- Baseline assessment

In each country, a pre-intervention retrospective data collection will take place in a sub-set of project sites.

This data collection activity will:

1. Capture data that will answer the TB service indicators and clinical outcomes listed in the primary objectives.
2. Capture key data points that will be needed to estimate project targets

The main objectives of the CaP TB interventions are:

- to increase the number of children diagnosed with TB
- To increase the number of children who are started on and complete DS-TB treatment
- To increase the number of children who are started on and complete TB preventive therapy.

Key indicators that will be used to monitor project interventions and assess impact will therefore be:

- Number of pediatric cases diagnosed with active TB disease
- Number and proportion of pediatric DS-TB cases who achieve treatment success
- Number and proportion of pediatric patients started on preventive therapy of those who are eligible for preventive therapy
- Number and proportion of pediatric patients on preventive treatment who completed therapy

Data sources for these indicators during the pre-intervention phase are described in Table 1.

In each site, trained data collectors will retrospectively extract data from appropriate registers, logs, and in some cases patient files, for a period of 12 months starting 6 or more months before the start date of data extraction. This time frame has been selected in order to allow to accurately measure indicators on treatment completion for both drug-sensitive TB treatment and preventive therapy (treatment lasts 6 months in both cases). In certain cases, the data sampling frame will start more than 6 months before the data extraction if CaP TB interventions had started during the sampling frame. Thus the sampling frame was chosen so that the end date of retrospective pre-intervention data abstraction would be before the start of any intervention to ensure that the baseline data was not affected by the early activities. For some indicators, baseline data will not be available in existing registers or logbooks. Data collectors will extract data from all pediatric entry points within the facility, including MNCH, HIV, nutrition and other relevant pediatric health care entry points. Only required variables will be abstracted and entered into the password-protected computers. Data with the patient date of birth (DOB), patient ID (if any), TB treatment ID and preventive treatment ID will be collected. The patient ID (if any), TB treatment ID and preventive treatment ID will all be encrypted before being uploaded into the CaP TB Database Entry Tool. DOB will be abstracted in order to facilitate tracking across registers if needed, as well as for data quality assurance purposes. This information will only be used to link registers. Only de-identified patient data will be abstracted into our global project

database. No other unique identifiers such as contact details of the children or their caregiver will be abstracted from the registers. Data from included pediatric patients will be extracted from the registers and recorded in the CapTB Baseline Data Abstraction form from where the data will be entered into a CaP TB Database Entry Tool (Excel-based and/or Web/mobile App-based) developed for pre-and post-intervention data , in a password protected computer and/or Android device (See Annex A: CapTB Information System).

The target population for the pre-intervention assessment is the records of any pediatric patient (0-14 years of age) who attend one of the sub-set of pilot sites in any of the 10 CaP TB countries who receive TB services. TB services include:

- Screening for TB (clinical symptoms or signs)
- Receiving services (such as contact tracing) as a result of being a contact of a TB index case
- Clinical diagnostic assessment for TB
- Microbiologic diagnostic assessment for TB
- Treatment for active TB
- TB preventive therapy

Data collection of pre-intervention baseline data will take place prior to the start of CapTB interventions or immediately after, ensuring that the calculated data abstraction period does not overlap with any of the early project interventions. Data collection in each site may last up to 4 days and in some cases data collection may last several days longer.

VI. Evaluation Design and Methods

CaP TB implementation data will be collected by project-specific data collection tools and/or from existing registers. Whenever possible, the CaP TB data collection will use existing site level data collection tools to gather the data for the project. However, where such tools do not exist or the tools have gaps to capture all the necessary indicators, we will introduce project-specific data collection tools, the Pediatric TB Intensified Case Finding Screening Tool (ICF tool), the CaP TB Pediatric TB Form and any other form that may evolve as we learn lessons from project implementation.

Use of project-specific data collection tools
As part of the CaP TB intervention, healthcare workers will fill out a Pediatric TB Intensified Case Finding Screening Tool (ICF tool). This tool has no identifying information and will provide information on 1) number of children screened for TB in each healthcare entry point (e.g. pediatric ward, outpatient department, MNCH clinic, and nutrition clinic); 2) sex of the child; 3) age of the child; 4) if the child has any signs of symptoms concerning for TB (according to WHO guidance); 5) if the child is a contact of a TB case; and 6) the date of screening. The forms are also color coded according to the healthcare entry points where children are seen (White= MCH/PMTCT; Blue= outpatient department; Pink= Nutrition; Yellow= Pediatric Ward, Other=to be decided). See Attachment: Pediatric TB Intensified Case Finding Screening Tool.

For children with presumptive TB: For all patients with a positive TB screen (children who have signs and symptoms concerning for TB according to the left side of the ICF tool), or for children who are a household contact of a TB case, the CaP TB Pediatric TB Form will be used to collect data on TB clinical and bacteriological investigations, as well as treatment initiation information at patient level. In addition, this form will be used directly to screen for TB symptoms in HIV clinics and during screening of contacts of TB index cases in the community, instead of the ICF tool for those 2 entry points, in effort to minimize unnecessary duplication of data entry. This CaP TB Pediatric TB Form will be adapted to country-specific requirements in collaboration with the MOH. This form will be printed on quadruplicate carbonated multi-colored papers (See Attachment C: CapTB Pediatric Form).

After the child has a positive TB screen the appropriate healthcare worker, such as the treating clinician or TB nurse, will begin filling out their section designated for requesting health workers on the CaP TB Pediatric TB Form, pre-printed with sequential numbers in each country using the Form. For children screened at HIV clinics or during contact tracing, this section will be directly filled for all children screened, irrespective of the presence of signs or symptoms or TB. This part of the form contains identifying information of the pediatric patient, contact information and identifies if the patient is exhibiting sign or symptoms concerning of TB, and if so which ones. If signs or symptoms of TB are identified, the facility TB nurse or focal point will then detach the last copy of the form (the pink form) and keep this form at the facility. The remaining three carbonated copies of the CaP TB Pediatric TB form will then be used as a referral form for sample collection and TB testing. The patient or patient's caregiver will be sent with the remaining three copies of the carbonated form to have a specimen collected. The healthcare worker collecting the sample will then fill out the section "To be filled at specimen collection site." This section contains information on the date of collection and type of specimen collected. The remaining three copies of the carbonated form, along with the specimen will be sent to the laboratory for specimen processing. The laboratory staff will fill out the section "To be filled by lab personnel." This section will contain information about the type of testing performed, the date of testing and the results of testing. The laboratory staff will detach one copy (the blue form) and keep this at the laboratory. The remaining two copies of the CaP TB Pediatric TB form, which contains the patient's results, will then be returned to the requesting facility. The requesting facility/treatment unit will then complete the section "To be filled by TB treatment unit." This section will contain additional information, including results of chest X-ray and if the patient was diagnosed based on clinical symptoms with or without radiologic signs. This section will also contain information about treatment initiation or the facility to which the patient was referred for treatment initiation as well as the patient's TB treatment register number. The first (white) page will be kept at the requesting facility for their records.

The CaP TB Pediatric TB Form will also be used for referral for an initiation of TB preventive therapy, primarily during contact tracing or screening at HIV clinics. The relevant healthcare worker will fill the section "To be filled by requesting health worker" and the section "To be filled during contact tracing and in HIV clinic only." These sections contain identifying information of the pediatric patient, contact information and information on eligibility for TB preventive therapy. If the child was identified at the HIV clinic, the healthcare worker keeps the last copy (pink) and the child/caregiver will bring with him/her the three remaining copies of the carbonated form and gives them to the unit initiating PT. If the child was identified through

contact tracing in the household, the community health worker (CHW) gives the last copy (pink) to the patient or patient's caregiver and the CHW brings the remaining 3-copy form to the TB unit. When the patient presents to the facility for TB preventive therapy initiation, the healthcare worker initiating TB preventive therapy will complete the section "To be filled by HCW providing PT." This section contains information on TB preventive therapy initiation, date of initiation and the patient's preventative therapy register number. In the event that the initiation date is not recorded, an EGPAF data collector will be responsible for following up with the entry point or facility providing TB preventative therapy. Upon completion of the form, one copy will remain at the facility (1st page) and the data entry copy (2nd page) will be transferred to the EGPAF office for entry into the project database.

CaP-TB data collector will ensure timely data collection and reporting from all CaP-TB sites. This copy is the project's data collection form. The form that data collectors will be collecting has all identifying information for the patient blocked out, except the child ID (if any), the TB treatment register number (or TB preventive therapy register number), the sex, and the date of birth to protect patient confidentiality.

Data collectors will also collect the ICF tools from the various entry points and collate aggregated data using an ICF data abstraction tool (Attachment B: Pediatric TB Intensified Case Finding Screening Data Abstraction Tool) which will be entered directly into the electronic data entry form. Data collectors will also extract information, such as date of treatment completion, from the TB treatment and TB preventive therapy registers. In the pilot phase (years 1 and 2), data collectors will visit sites frequently for data extraction (approximately every 4-6 weeks). In the expansion phase (years 3 and 4), data collectors will visit sites less frequently for data extraction (approximately every 1-3 months).

Data collection when the project-specific data collection tools are not used
If the CaP TB Pediatric TB Form is not completed or the country does not support use of the ICF Tool and/or CaP TB Pediatric TB Form for all or part of the data collection, data collectors will extract data from relevant sources such as existing registers and logs as in the pre-intervention baseline data collection phase. An indicator mapping exercise will first be conducted to identify the location and source of all required data. This indicator mapping exercise is expected to inform the data collection process which will take place on site, at the project facility. Data will be extracted from the source register or log and, recorded in the baseline data abstraction tool and entered into the CaP TB Database Entry Tool developed for pre-and post-intervention data. Only data required for the project M&E activities will be extracted and will be the same data collected through the CaP TB Pediatric TB Form. EGPAF will closely monitor data collection activities of any country team that cannot use the CaP TB Pediatric TB Form to ensure high quality data is collected. All data collection tools and processes will be approved by the in-country IRB.

A. Table 1. Indicator Outcomes and Data Sources

This table presents an example of data sources. Specific data sources may vary from country to country. All country-specific data sources will be approved for data extraction by the local IRB.

Primary Outcomes	Data Source Pre-Intervention	Data Source Post-Intervention
Number and proportion of children screened for TB among clinic attendees	TB screening logbooks/registers	Pediatric TB Intensified Case Finding Screening Tools and Clinic attendance registers<br><br>And/or<br><br>TB screening logbooks/registers
Number and proportion of presumptive pediatric TB cases identified among children screened for TB	TB screening logbooks/registers Presumptive TB registers	CaP TB Pediatric TB Form<br><br>And/or<br><br>TB screening logbooks/registers
Number and proportion of presumptive TB cases referred for lab-based TB diagnosis	TB screening logbooks/registers or Presumptive TB registers	CaP TB Pediatric TB Form<br><br>And/or<br><br>TB screening logbooks/registers, Presumptive TB registers
Number and proportion of pediatric (0-14 years) presumptive TB cases who are tested with Xpert	TB diagnostic logbooks/registers or Xpert logbooks/registers	CaP TB Pediatric TB Form<br><br>And/or<br><br>TB diagnostic logbooks/registers or Xpert logbooks/registers
Number and proportion of pediatric TB cases diagnosed with active TB disease among presumptive TB cases	Presumptive TB registers and TB registers	CaP TB Pediatric TB Form<br><br>And/or<br><br>TB registers/logbooks
Time between when a child is identified as a presumptive TB case and when the child is diagnosed with TB	Presumptive TB registers, TB registers/logbooks	CaP TB Pediatric TB Form<br><br>And/or<br><br>Presumptive TB registers, TB registers/logbooks
Number and proportion of pediatric TB cases started on DS-TB treatment	TB registers/logbooks	CaP TB Pediatric TB Form<br><br>And/or<br><br>TB registers/logbooks
Time between when a child is identified as a presumptive TB case and when the child is initiated on TB treatment	Presumptive TB registers, TB registers/logbooks	CaP TB Pediatric TB Form<br><br>And/or

		Presumptive TB registers, TB registers/logbooks
Number and proportion of pediatric DS-TB cases who achieve treatment success	TB treatment register	TB treatment register
Number and proportion of all TB index cases for whom successful contact tracing has been done	TB registers/logbooks<br>Contact tracing forms and registers if available	CaP TB Pediatric TB Form and TB register<br><br>And/or<br><br>TB registers/logbooks<br>Contact tracing forms and registers if available
Number and proportion of pediatric household contacts who are negative to TB screening	TB registers/logbooks<br>Contact tracing forms and registers if available	CaP TB Pediatric TB Form<br><br>And/or<br><br>TB registers/logbooks<br>Contact tracing forms and registers if available
Number and proportion of screened negative pediatric contacts eligible for preventive therapy	TB registers/logbooks<br>Contact tracing forms and registers if available	CaP TB Pediatric TB Form<br><br>And/or<br><br>TB registers/logbooks<br>Contact tracing forms and registers if available
Number and proportion of pediatric patients started on preventive therapy of those who are eligible for preventive therapy	TB preventive therapy register	CaP TB Pediatric TB Form<br><br>And/or<br><br>TB preventive therapy register
Number and proportion of pediatric patients on preventive treatment who completed therapy	TB preventive therapy register	TB preventive therapy register<br><br>And/or<br><br>TB preventive therapy register

B. Table 2. Additional Indicator Outcomes and Data Sources

Additional data will be collected during the pre-intervention phase and will be used to estimate project targets. This table presents an example of data sources. Specific data sources may vary

from country to country. All country-specific data sources will be approved for data extraction by the local IRB.

Primary Outcomes	Data source
Number of HIV positive children (0-14 years) in care	HIV clinic register
Number of adult TB cases notified (disaggregated by age, sex, type of TB [PTB smear positive, PTB smear negative; EPTB], new or previously treated case, DS or MDRTB case)	TB treatment Register
Number of pediatric household contacts recorded for each adult TB case (disaggregated by age, sex)	TB register or TB contact tracing register<br>In countries where information is not recorded in the above described register, there might be the need to extract this information from a sample of patient forms
Number of HIV positive pregnant women in care (disaggregated by age)	PMTCT register
Number of HIV+/TB pregnant women (disaggregated by age, type of TB [PTB smear positive, PTB smear negative; EPTB], new or previously treated case, DS or MDRTB case)	PMTCT register

VII. Use of data generated through CaP TB program evaluation to perfom secondary analysis

A. Use of CaP TB Program evaluation data to inform cost-effectiveness analysis

Data documenting the costs and cost-effectiveness of pediatric TB interventions remains limited[XXXV],[XXXVI], and this represents one of the contributing factors to the under-funding of childhood TB programming [XXXVII]. The data generated through the TIPPI evaluation are well placed to inform the cost-effectiveness assessment of the package of pediatric TB interventions implemented under the CaP TB project.
To this aim, EGPAF will sub-contract University of Sheffield, key leader in childhood TB health economic modelling, to perform a cost-effectiveness analysis (CEA).The resulting effects estimates will be based on routinely collected project intervention country-level aggregates from key indicators describing the cascade of care, as well facility-level data for the comparison of the pre- and post- project intervention phase. All data to be used will have already been collected through TIPPI Pre-Intervention evaluation (described in section V, subsection A) and CaP-TB implementation evaluation (described in section V, subsection I).
Only aggregated clinical data will be shared with University of Sheffield and thus considered for the CEA analysis, as described below:

i. Averaged monthly rates per site (including absolute numerator and denominator) of pediatric (children aged 0-14 years) TB notifications, DS-TB initiations, and PT initiations for a subset of sites for which both baseline and prospective data have already been collected (restricting to sites where data on both periods are available).
ii. Country-aggregated project intervention numbers of: a) Children screened for TB , b) Children identified as presumptive TB, c) Children with presumptive TB tested with Xpert, d) Children diagnosed with TB, e) Children treated for DS- TB, f) All TB index cases for whom successful contact tracing has been done, g) Children started on TB Preventive Therapy (disaggregated by HIV and Contact investigation entry points), and h) Sites contributing to the country-level dataset.

All patient-level indicators mentioned in points i and ii above are included in Table 1. The cost-effectiveness analysis will use pre-intervention and project intervention data, with the potential for sub-analysis of shorter targeted period during the intervention phase.
The final cost-effectiveness analysis outcomes are expected to be reported by country, and individual countries will be identified to allow to use context-specific results to inform decision-making.

The costs will be estimated as follows:

- Core health services costs will be derived from published literature.
- CaP TB intervention costs will be estimated based on EGPAF CaP TB budgets and expenditure data from EGPAF's financial system.
- EGPAF country team staff will assist in allocating cost items to specific activities within the CaPTB cascade of service delivery.

B. Use of TIPPI data to assess the impact of COVID 19-related measures on accessibility and delivery of pediatric TB services

The COVID-19 pandemic and the measures enforced by governments to control it have affected health services in general, and the repercussions on TB services have been estimated to be significant[XXXVIII]. However, evidence on the impact on pediatric TB services is scarce. CaP TB project data collection processes could continue throughout the crisis caused by the COVID-19 pandemic. Therefore data collected under the TIPPI protocol could be utilized to analyze the impact that the measures enforced by governments to control the COVID-19 pandemic had on the accessibility and delivery of pediatric TB services.

Below is a summary of the analyses that we would perform to assess this impact:

- Measure the changes in number of pediatric patients seeking care at facility, through uninterrupted time series of variations in the number of clinic attendees
- Measure the changes in quality determinants of pediatric TB services offered before and during the COVID-19 pandemic throughout the cascade of care. To measure such changes, we will run uninterrupted timeseries analysis comparing number and proportions of most of the TB services-related indicators described in this protocol

Only routine protocol data will be collected as per the description of indicators, data collection tools, data gathering and data management activities described elsewhere in this document.

VIII. Data Gathering Activities

A. Table 3. Study Data Gathering Activities During Pre-intervention and CaP TB Intervention Phases

**Data Gathering Activities**	**Pre-intervention evaluation**	**CaP TB evaluation**
	Retrospective data collection in a sub-set of project sites (15-20 pilot sites per country)	Periodic data collection in all project sites
Study population	Pediatric patients (0-14) who attend a CaP TB pilot site who receive TB services	Pediatric patients (0-14) who attend a CaP TB site who receive TB services
Sample size	10-20 purposively selected pilot sites in each country, for a total of up to 200 sites across all ten countries. Sites will be selected based on geography (urban vs rural) and health facility level. We estimate that 12 months of data will be sufficient to appropriately assess the indicators from the primary objectives under standard of care conditions. The overall sample size in each country and per facility sample size will vary greatly by facility level, pediatric population density and indicator. However, we estimate that we will collect data for at least 5 children with presumptive TB in each clinic.	Up to 70 sites in each country for a total of up to 700 sites across all ten countries.
Location of activity	15-20 CaP TB pilot sites in each country	All CaP TB sites across 10 countries (up to 700 sites)
Timing	Retrospective data collection will occur upon site selection. It is estimated that the retrospective data collection for all sample project sites in a given country will take six to ten weeks.	Data collection will begin when each site begins implementation of CaP TB and will go on through the end of the project
Method	Data abstraction of TB registers, HIV registers, PMTCT registers and other relevant logbooks	Data abstraction from clinic attendees registers, Pediatric TB Intensified Case Finding Screening Tool, CaP TB Pediatric TB Form, TB treatment register, TB preventive therapy register and other TB registers and logbooks as needed

Informed Consent documents	Informed consent waiver to be requested	Informed consent waiver to be requested
Data collection tool	Baseline data abstraction tool	Pediatric TB Intensified Case Finding Screening Data Abstraction Tool and CaP TB Database Entry Tool

IX. Data Management and Statistical Analysis

Quantitative de-identifiable data extracted from the source documents will be coded and entered directly into a database that will be designed specifically for the project and stored on a secure cloud server. Data will be aggregated in the database and entered into an electronic dashboard that will demonstrate the data standardized to project indicators and data elements. Frequencies will be calculated for each response and descriptive statistics will be calculated.

Project data sample size: As this is a project evaluation, no specific sample size has been calculated. However, we estimate that the project will increase case detection by 2-fold and result in an additional 16,471 pediatric TB cases being treated.[XXXIX] This is based on an assessment of the number of children we project to screen (both HIV positive and HIV negative) in our project sites throughout the project. From the literature, we estimate that approximate Number Needed to Screen (NNS) to identify 1 TB case is 41 children. Assume that Number Needed to Screen (NNS) to identify 1 TB case is 41 (estimated NNS when symptom based screening is the only primary screening done)[XL,XLI]
Preventive treatment coverage is estimated to increase by four-fold, with an additional 52,317 children to be initiated on preventive therapy. This is based on the estimated number of child TB contacts we will identify through contact tracing plus HIV-infected children who qualify for preventative treatment.

To compare pre- and post-intervention outcomes, we will use either the ratio of the two proportions (relative risk) or the difference between the two proportions (risk difference). We will use Z-tests to assess the statistical significance of the estimated effect of CaP TB.

For measuring turn-around-time (TAT) between when a child is identified as a presumptive TB case and when the child is diagnosed with TB and the TAT between when a child is diagnosed as a presumptive TB case and when the child initiates TB treatment, if the TATs are approximately normally distributed, we will use the paired t-test to assess for statistical significance, otherwise we will use the Wilcoxon rank sum non-parametric test.

A. Quality Assurance

All data collectors will be trained in quantitative data collection by EGPAF staff. Prior to training, all data sources, abstraction and collection tools and procedures will be reviewed and tested. The HQ EGPAF team will provide on-going support to in-country teams throughout program implementation and all data collection activities.

All data from project tools and registers will be entered into a database that will be designed specifically for the project and stored on a secure web-based server. The database will allow data quality checks on a routine basis throughout the data collection period to ensure that data are within a feasible range. For instance, data values related to dates must be inferior to the actual database upload dates, and superior to dates of birth, etc. in order to be coherent with the cascade of care. Any out of range values will be verified by tracing data through the source document during the data collection visits described below, and by contacting the facility for further discussion and resolution.

Data quality will be ensured by training EGPAF staff in-country and comprehensive documentation of data collection processes and procedures prior to the collection of data. In addition to training and documentation of data collection procedures, quality assurance rounds will be conducted by in-country EGPAF staff during data collection at randomly selected sites to ensure proper completion of the project forms (the ICF tool and the CaP TB Pediatric TB Form). Quality assurance rounds will assess for completeness and consistency to check for anomalous data recording, omission and extraction. Data will be reviewed by CaP TB project facility focal person and in-country EGPAF staff who will undergo data quality assurance training. In addition, the project Country Implementation Manager and M&E focal person will routinely review the data for completion and accuracy, comparing a randomly selected sample of CaP TB Pediatric TB forms with data entered into the database. Corrections will be made as necessary and data collection and entry clerks re-trained if needed. The Washington-based Senior Strategic Information and Evaluation (SIE) Officer and HQ SIE backstops will receive de-identified data on a regular basis for additional data validation, to ensure completeness of data. The HQ Senior SIE Officer and project M&E team will be responsible for maintaining the integrity of the in-country project database. The project will design and implement strict data quality and management SOPs and a tracking system for documentation of all entries, changes, or updates to the project database.

As with pre-intervention aggregated data, post-intervention aggregated data will be subject to data quality verification processes.

X. Privacy and Confidentiality

Aggregated data will not contain any identifiable information about clients and there is minimal potential for a breach in confidentiality. Additionally, patient level data abstracted for pre and post-intervention analysis will be aggregated for dissemination of findings to further protect confidentiality and will not contain any identifiable information about patients. The CaP TB Database Entry Tool will be located on a password-protected computer and/or Android device. The name of the patient and their caregiver, contact details and other identifiable information such addresses, phone numbers will not be entered into the CaP TB Database Entry Tool at any time.

Only the health facility staff will have access to the identification number that links the ID to the patient's name and other identifiable patient information such as caregiver's name and address, TB Prevention therapy number, TB treatment number and patient identification numbers (if available) will be automatically encrypted before upload to the global database, so that upon review at headquarter level, an evaluation identification number is assigned to all children.

However, to protect the confidentiality of the patient, we will implement the following protective measures:

a) At the country level, patient information, including the patient ID (if any) and treatment register numbers will be entered into the country CaP TB Database Entry Tool, and will be automatically encrypted, with the exception of DOB, before uploaded into the project database.
b) The database with global data is housed in Microsoft cloud. The database developers, the Diagnostics Advisor and the Senior Strategic Information and Evaluation Officer and other key relevant staff such as the Senior Biostatistician responsible for data analysis, have access to all the country files, globally. These are the files that contain the patient IDs. Access to the database is password protected and access to this file can only be provided by the database developer.
c) All calculations are done on the backend of the database system, including the count of unique number of patients; the dashboard which is the project's data visualization tool, presents aggregated data without patient IDs or treatment register numbers.
d) The CaP TB Pediatric Form ID Number, generated by the project, will be made visible and will be used to track records with data quality errors.
e) Permission must be given to access the global dashboard by either the database developer or the Senior Strategic Information and Evaluation Officer to the project. Permission is granted to key EGPAF project staff only, such as country implementing managers, M&E staff and principal investigators.
f) The dashboard is password protected and only EGPAF employee, with an EGPAF email can be granted access to the global dashboard.
g) Patient and treatment register IDs will not be included in any analysis or dissemination of results.

As new security measures become available to protect the data in the database and the dashboard, they will be implemented as possible. Whenever feasible, an agreement on data sharing policy or practices will be established between EGPAF and participating country.

All computers used for data entry and analysis will be password protected. Once at EGPAF offices, computers will be in a locked cabinet, in a locked office with limited access in each project country. Only relevant members of the project team in country will have access to these data for entry, quality audit, analysis and reporting purposes.

Facility based data abstraction and any discussions about data quality, patient care and management will be conducted in private areas free from general view and out of hearing range as much as possible.

Data presented as part of study findings in papers or at conferences will not include any participants' names, IDs or any other identifiable information. By virtue of their presence in the clinic and data abstraction and quality improvement activities from existing clinic registers and additional sources, project staff will view records of patients attending the various clinic units, including those not enrolled in the evaluation. However, individual data from non-study participants will not be abstracted/collected for evaluation purposes. All project staff will receive ethics training to ensure compliance with human subject research requirements.

Following the completion of the evaluation and all analyses, paper-based documents for all data collection activities, including the second yellow copy of the Cap TB form, will be stored for three years and then destroyed. The first white copy of the form will stay at requesting health facilities and will be kept according to local regulations. Key patient information from the CaP TB form will also have to be recorded by the site personnel on NTP forms, registers, and patient files.

XI. Ethical Considerations

A. IRB Review

This protocol will be reviewed by an Institutional Review Board (IRB) in the US and an ethical review committee in each of the ten project countries. The local ethics committees will also receive country-specific details about the evaluation. Participants will not be compensated.

B. Ethics Training

All EGPAF data collectors, monitoring and evaluation staff and management staff will be trained in research ethics prior to starting data abstraction and collection activities.

C. Conflict of Interest

The PIs and co-investigators listed on this protocol have no affiliations with or involvement in any organization or entity with any financial interest (such as honoraria, grants, employment, consultancies), or non-financial interest (such as personal or professional relationships, affiliations, knowledge or beliefs) in the subject matter or materials discussed in this protocol.

D. Informed Consent

The pre- and post- intervention, quantitative data abstraction is limited to data that are routinely documented as part of standard medical care and/or program services. The data will be abstracted from CaP TB project forms (Pediatric TB Intensified Case Finding Screening Tool or CaP TB Pediatric TB Form), registers, logbooks or rarely, patient files. With the exception of the patient ID (if any), the TB treatment register number or the Preventative Therapy register number, no identifying information will be collected. This information will be encrypted before upload to the database. Only the health facility staff will have access to the identification number that links the ID or treatment register number to the child's name and other identifiable patient information such as caregiver's name and address.

A waiver of informed consent is being requested for all patients whose data will be abstracted for the following reasons according to the U.S. Code of Federal Regulations (CFR 46.116(d)) [XLII]:

1. "The research presents no more than minimal risk of harm to subjects;

2. The waiver or alteration will not adversely affect the rights and welfare of the subjects; and
3. The research could not practically be carried out without the waiver or alteration"

E. Risk and Benefits to Participants

The pre- and post-intervention, quantitative data being abstracted for evaluation purpose are identifiable but it poses no more than minimal risk to pediatric patients receiving TB services and it is no greater than what they would experience during routine provision of these services. Potential, minimal risks include the unintended disclosure of TB status of children if the child is known to the data abstractor during data abstraction activities. The interventions the CaP TB project supports utilize quality-assured and WHO-recommended drugs and diagnostics that are in use in country and/or are part of country guidance and thus we view the risks of the CaP TB project itself to be of no greater risk to participants than that of receiving standard healthcare in the project countries.

Potential benefits to participants include access to improved pediatric TB care, including improved access to WHO-recommended diagnostics and treatments. Potential benefits to society include identifying and resolving problems in the quality of pediatric TB care services sooner, providing information to MOH, other implementing partners and donors that may help to plan and prioritize more effective pediatric TB services. These improved services may lead to reduced morbidity and mortality associated with pediatric TB.

F. Follow-Up

Beyond standard care and treatment provided by CaP TB and the MOH, no additional follow up of patients is planned in this evaluation.

XII. Dissemination of Results and Publication Policy

Data abstracted or collected for this evaluation will be used to 1) Estimate project targets; 2) evaluate the effect of CaP TB as compared to standard of care pediatric TB services as well as to 3) assess the effects of CaP TB during the continued scale up phase (years 3 and 4). Data will be abstracted/collected, analyzed and reviewed on a routine basis to inform program implementation and quality improvement activities, target setting and adjustments, internal and external reports, abstracts, presentations and international guidelines.. Findings will be shared across the project countries, with MOHs, with other implementing partners, with Unitaid and the WHO and the international community through semi-annual reports to the funder (Unitaid), grey literature, and publications in peer-reviewed journals, meetings and presentations. In order to perform cost-effectiveness analysis, aggregated clinical data will be abstracted and shared with University of Sheffield. Unpublished data analysis and databases might be shared with WHO and with WHO's collaborators that will be commissioned to compile and assess the evidence needed to support revisions of guidelines and policy formulation. Findings will also be shared through EGPAF data dashboards and website.

Data presented as part of study findings in papers or at conferences will not include any participants' names, IDs or study identifiers. Only the names of project countries will remain identifiable if required.

XIII. Problems Anticipated

Implementation of the CaP TB project is dependent on the approval and continued support of the country MOHs and National TB Programs (NTPs). At the time of protocol drafting, the CaP TB project has been presented to the MOHs and National TB Programs in all ten countries and all country MOHs and NTPs are supportive. However, national MOHs and National TB Programs may withdraw support at any time which may affect the overall duration of the project. The project is also dependent on the national procurement and supply chains to maintain an adequate supply of appropriate drugs and diagnostics. While the CaP TB project includes activities to strengthen procurement and supply chain systems, drug or diagnostic stock outs may occur. However, as this is an evaluation of a pragmatic program implemented in a "real-world" setting, documenting stock shortages or stock outs will be important to evaluating the feasibility of our project. Finally, political instability or other instability may interrupt project activities or data collection. At this point, project areas in all ten countries are sufficiently stable to allow for project activities and data collection, and the project and country team will continue to monitor the situation and plan accordingly.

XIV. Project Management

The Elizabeth Glaser Pediatric AIDS Foundation is responsible for protocol development and other study documents, overall conduct and management and the scientific integrity of the study. EGPAF will also serve as the liaison for communication and coordination with ethical review boards, the Ministry of Health in each project country, UNITAID and other stakeholders. The overall project management, including all aspects of the intervention, will be led by Dr. Jennifer Cohn, Senior Director of Innovations at EGPAF. The day-to-day management of the project's implementation will be managed by Mikhael de Souza, Project Director of CaP TB and Martina Casenghi, Technical Director of CaP TB. The data and program evaluation will be managed by Shirin Kakayeva, Strategic Information and Evaluation lead for the CaP TB project. Dr. Rhoderick Machekano is a Senior Biostatistician at EGPAF and will be responsible for developing of the statistical analysis plan, review and analysis of data, and contribute to the write up of study findings. Each project country has a country implementation manager who will be responsible for the day-to-day management of activities, and a Senior Strategic Information and Evaluation officer who will manage day-to-day responsibilities for the project evaluation.

XV. Annex A: CaP TB Information System

The CaP TB Database Entry Tool will abstract the information contained on, and matching the 2nd page (blinded for patient personal details) of the CaP TB Pediatric TB Form. We will use a combination of web/mobile App or Excel entry templates, both directly feeding into a unique database. Below are example of data flow in both models. A generic Excel based template of the CaP TB Database Entry Tool is provided as part of this protocol submission. As the CaP TB Pediatric TB Form will be adapted by countries to align with country-specific requirements, the CaP TB Database Entry Tool will also be consequently adapted to a perfect match.

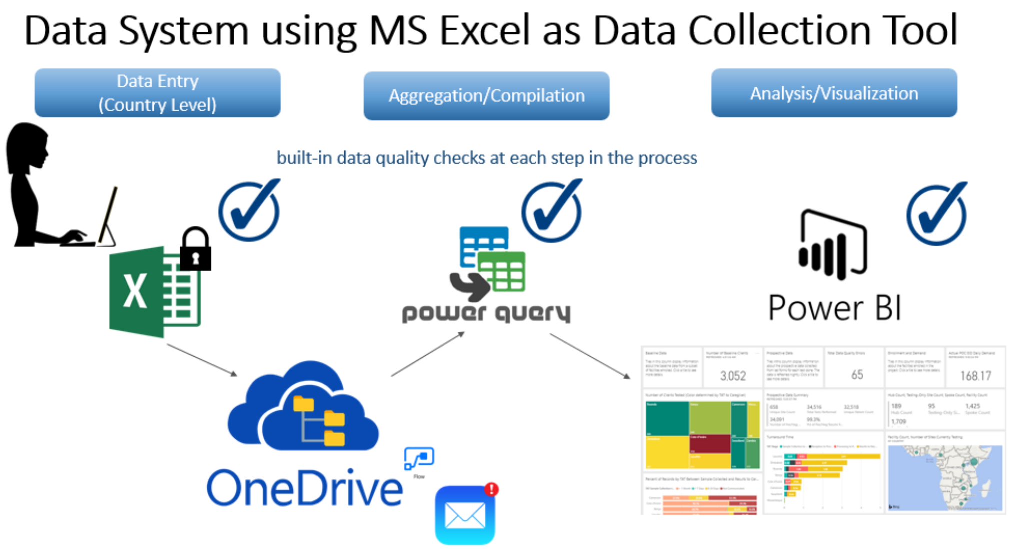

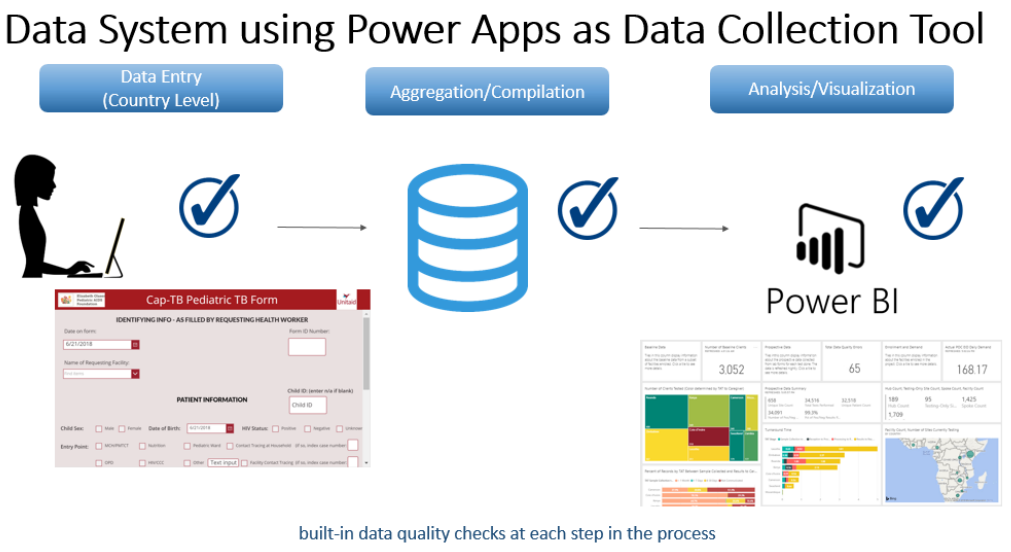

References

[I] TB Facts. http://www.tbfacts.org/tb-children/#sthash.F7e8OM9G.dpuf
[II] Seddon et al 2015_Counting children with TB_ IJTLD
[III] Global tuberculosis report 2017. Geneva: World Health Organization; 2017. Licence: CC BY-NC- SA 3.0 IGO.
[IV] WHO Global TB report 2015
[V] Marais BJ et al. The natural history of childhood intra-thoracic tuberculosis: a critical review of literature from the pre-chemotherapy era. INT J TUBERC LUNG DIS 8(4):392–402.
[VI] Tiemersma EW, van der Werf MJ, Borgdorff MW, Williams BG, Nagelkerke NJD (2011) Natural History of Tuberculosis: Duration and Fatality of Untreated Pulmonary Tuberculosis in HIV Negative Patients: A Systematic Review. PLoS ONE 6(4): e17601. doi:10.1371/journal.pone.0017601
[VII] Mwangwa F et al. Gaps in the child tuberculosis care cascade in 32 rural communities in T Uganda and Kenya. J Clin Tuberc Other Mycobact Dis 9 (2017) 24–29.
[VIII] "Global Tuberculosis Report", WHO, Geneva, 2016 www.who.int/tb/publications/global_report/en/
[IX] WHO Global TB report ; Seddon and Shingadia 2014 http://www.ncbi.nlm.nih.gov/pmc/articles/PMC4069045/
[X] Jeena PM et al, Int J Tuberc Lung Dis 2002
[XI] Schaaf HS et al, BMC Infect 2008
[XII] WHO Global TB report 2016. Table 3.7
[XIII] Pediatric Int Child Health. 2012 Nov;32 Suppl 2:S30-7. Doi: 10.1179/2046904712Z.00000000076.
Tuberculosis diagnostics for children in high-burden countries: what is available and what is needed. Cuevas LE1, Petrucci R, Swaminathan S
[XIV] Brigden G et al. Getting it right for children: improving tuberculosis treatment access and new treatment options. Expert Rev Anti Infect Ther. 2015:1-11.
[XV] WHO. Country tuberculosis profiles, India. Available at https://extranet.who.int/sree/Reports?op=Replet&name=%2FWHO_HQ_Reports%2FG2%2FPROD%2FEXT%2FTBCountryProfile&ISO2=IN&LAN=EN&outtype=html Accessed on 23 December 2016.
[XVI] Hazarika. Role of Private Sector in Providing Tuberculosis Care: Evidence from a Population-based Survey in India. J Glob Infect Dis. 2011; 3(1):19-24.s
[XVII] WHO. End Tb strategy. Available at http://apps.who.int/gb/ebwha/pdf_files/EB134/B134_12-en.pdf?ua=1. Accessed on 12 April 2017.
[XVIII] Global Plan to stop TB 2016-2020
[XIX] Roadmap for childhood tuberculosis: towards zero TB deaths
[XX] Detjen A et al, Systematic review on Xpert for pediatric TB.
[XXI] Kebede S et al. Intensive case finding of children using the MCH approach. Presentation at the Annual meeting of the Childhood TB Working Group. The Union Conference 2016. Available at http://www.who.int/tb/areas-of-work/children/SenaitKebede_ICFEthiopia.pdf?ua=1.
[XXII] Greig J et al. Similar Mortality and Reduced Loss to Follow-Up in Integrated Compared With Vertical Programs Providing Antiretroviral Treatment in Sub-Saharan Africa. JAIDS. 2012; 59(5): e92-e98.
[XXIII] Uyei J et al. Integrated delivery of HIV and tuberculosis services in sub-Saharan Africa: a systematic review. Lancet Infect Dis 2011;11: 855–67.
[XXIV] Penazzato M et al. Development of a global research agenda for pediatric & adolescent HIV. 9th IAS. Paris, France. July 22-26 2017.
XXV Latent tuberculosis infection: updated and consolidated guidelines for programmatic management. Geneva: World Health Organization; 2018. Licence: CC BY-NC-SA 3.0 IGO.
[XXVI] "Global Tuberculosis Report", WHO, Geneva, 2015 www.who.int/tb/publications/global_report/en
[XXVII] The policy and practice divide for childhood tuberculosis in Africa: a landscape analysis. March 2016 http://www.africanstrategies4health.org/resources/tuberculosis (accessed on May 2016)
[XXVIII] Stop TB Partnership Global Plan to End TB 2018-2022. The Paradigm Shift. 2019. Available at: http://www.stoptb.org/resources/publications/ (accessed on March 5th 2021)
[XXIX] Roadmap towards ending TB in children and adolescents. WHO, Geneva. 2018. Available at https://www.who.int/tb/publications/2018/tb-childhoodroadmap/en/. Accessed March 5th 2021
[XXX] The pediatric population is defined as children aged 0-14 years, consistently with WHO definition.
[XXXI] Lei et al. Public-private mix for tuberculosis care and control: A systematic review. International Journal of Infectious Diseases. 2014; 34:20-32.

[xxxii] GLI Planning for country transition to Xpert MTB/RIF Ultra Cartridges, WHO, Geneva, 2017 http://www.stoptb.org/wg/gli/assets/documents/GLI_ultra.pdf

[xxxiii] Latent TB Infection: Updated and consolidated guidelines for programmatic management, WHO, Geneva, 2018 http://www.who.int/tb/publications/2018/latent-tuberculosis-infection/en/

[xxxiv] WHO. Guidance for national tuberculosis programmes on the management of tuberculosis in children, 2nd Ed.

[xxxv] Mandalakas et al. Modelling the cost-effectiveness of strategies to prevent tuberculosis in child contacts in a high-burden setting.Thorax 2013; 68(3):247-55

[xxxvi] van Elsland SL et al. Cost-effectiveness of home-based vs. in-hospital treatment of paediatric tuberculous meningitis. Int J Tuberc Lung Dis. 2018; 22(10):1188-1195.

[xxxvii] Marais BJ et al. Tuberculosis in children, adolescents, and women. Lancet Respir Med. 2020; 8(4):335-337.

[xxxviii] McQuaid CFet al. The potential impact of COVID-19-related disruption on tuberculosis burden. Eur Respir J 2020; 56: 2001718.

[xxxix] As per WHO definition and drug labelling, the reformulated FDCs can only be administered to children <25kg. Children > 25 kg should be treated with the adult formulation.

XLKranzer K1, Houben RM, Glynn JR, Bekker LG, Wood R, Lawn SD.Yield of HIV-associated tuberculosis during intensified case finding in resource-limited settings: a systematic review and meta-analysis. Lancet Infect Dis. 2010 Feb;10(2):93-102

[XLI] Shapiro et al. A systematic review of the number needed to screen to detect a case of active tuberculosis in different risk groups Available at: http://www.who.int/tb/Review3NNS_case_active_TB_riskgroups.pdf

[XLII] US Code of Federal Regulations (46.116 (d); bolded by author):

(d) An IRB may approve a consent procedure which does not include, or which alters, some or all of the elements of informed consent set forth in this section, or waive the requirements to obtain informed consent provided the IRB finds and documents that:

(1) The research involves no more than minimal risk to the subjects;
(2) The waiver or alteration will not adversely affect the rights and welfare of the subjects;
(3) The research could not practicably be carried out without the waiver or alteration; and
(4) Whenever appropriate, the subjects will be provided with additional pertinent information after participation.

US Code of Federal Regulations (46.117; bolded by author):

> "(c) **An IRB may waive the requirement for the investigator to obtain a signed consent form for some or all subjects if it finds** either:
>
> (1) That the only record linking the subject and the research would be the consent document and the principal risk would be potential harm resulting from a breach of confidentiality. Each subject will be asked whether the subject wants documentation linking the subject with the research, and the subject's wishes will govern; or
>
> (2) **That the research presents no more than minimal risk of harm to subjects and involves no procedures for which written consent is normally required outside of the research context.**"
