# Supplementary material for: Improving TB detection among children in routine clinical care through intensified case finding in facility-based child health entry points and decentralized management: A before-and-after study in Nine Sub-Saharan African Countries
Source: PLOS Glob Public Health. 2024 Feb 5;4(2):e0002865. doi: 10.1371/journal.pgph.0002865 (PMC10843113; doi:10.1371/journal.pgph.0002865)
Supplement: S1 Acknowledgments — (PDF) [file pgph.0002865.s013.pdf]

CaP TB Study Team Members

Cameroon

Ministry of Health and National Tuberculosis Program
Dr. Ewos Guy
Dr. Kuaté Kuaté Albert

EGPAF Cameroon
Dr. Tchendjou Patrice
Dr. Leonie Simo
Dr. Nzima Nzima Valery
M. Simplice Lekeumo
M. Elvis Fru Moma

Cote d'Ivoire

Ministry of Health and National TB Program
Dr. Kouakou Jacquemin
Dr. Pongathié Adama Sanogo
Dr. Ehui Eboi, Dr Sidibé Souleymane

EGPAF Cote d'Ivoire
Dr.Diby Brou Charles Joseph
Dr. Kouadio Marc N'goran

Democratic Republic of Congo (DRC)

Ministry of Health and National TB Program
Dr. Michel Kaswa
Dr. Gertrude Lay
Dr. Judier Diala Diala
Dr.Grace Bolie
Dr.Nicole Anshambi
Dr. Patrick Tshey

EGPAF DRC
Dr. Aime Loando
Dr. Dieudonné Tshishi
Dr. Papy Ndjibu

In country partners
Dr. Nicolas NKIERE
M. Maxime Lunga

Kenya

Ministry of Health and National TB Program
Dr Gilchrist Lokoel
Gordon Odhiambo Okomo
Dr Jacqueline N. Kisia

EGPAF Kenya
Dr.Rose Masaba
Esther Wangui Kamau

<u>Lesotho</u>

Ministry of Health and National TB Program
Dr. Llang Maama
Manone Rantekoa

EGPAF Lesotho
Tseliso Marata
Dr. Zinga Kiuvu

<u>Malawi</u>

Ministry of Health and National TB Program
Dr. James Mpunga
Dr.Kuzani Mbendera
Lameck Mlauzi

EGPAF Malawi
Dr.Kwashie Kudiabor
Dr.Allan Ahimbisibwe
Cathy Golowa
Pilirani Banda

<u>Tanzania:</u>

Ministry of Health and National TB Program
Dr. Zuweina Kondo
Dr. Webhale Ntagazwa, Research Coordinator

EGPAF Tanzania
Dr. Sajida Kimambo
Dr.Frederick Haraka
Dr.Stella Kassone
Kassim Selemani
Dr.Roland van de Ven
Dr.Chrispine Kimario
Hassan Mattaka Mohamed

<u>Uganda</u>

Ministry of Health and National TB Program
Dr. Stavia Turyahabwe
Dr. Moorine Penninah Sekadde

EGPAF Uganda
Eliab Kajungu Natumanya

Dr. Edward Bitarakwate
Richard Okello
Henry Ijjo

<u>Zimbabwe</u>

Ministry of Health and National TB Program
Dr. Owen M. Mugurungi

EGPAF Zimbabwe
Dr. Agnes Mahomva
Emmanuel Tachiwenyika
